# Layer III pyramidal cells in the prefrontal cortex reveal morphological changes in subjects with depression, schizophrenia, and suicide

**DOI:** 10.1038/s41398-022-02128-0

**Published:** 2022-09-05

**Authors:** Nick Y. Larsen, Ninna Vihrs, Jesper Møller, Jon Sporring, Xueke Tan, Xixia Li, Gang Ji, Grazyna Rajkowska, Fei Sun, Jens R. Nyengaard

**Affiliations:** 1 Core Centre for Molecular Morphology, Section for Stereology and Microscopy, Department of Clinical Medicine, Aarhus University, Aarhus, Denmark; 2 Center of Functionally Integrative Neuroscience, Department of Clinical Medicine, Aarhus University, Aarhus, Denmark; 3 Sino-Danish Center for Education and Research, Aarhus, Denmark; 4 University of the Chinese Academy of Sciences, Beijing, China; 5 Centre for Stochastic Geometry and Advanced Bioimaging, Aalborg University, Aarhus University and University of Copenhagen, Aarhus, Denmark; 6 Department of Mathematical Sciences, Aalborg University, Aalborg, Denmark; 7 Department of Computer Science, University of Copenhagen, Copenhagen, Denmark; 8 National Key Laboratory of Biomacromolecules, CAS Center for Excellence in Biomacromolecules, Institute of Biophysics, Chinese Academy of Sciences, Beijing, China; 9 Center for Biological Imaging, Institute of Biophysics, Chinese Academy of Sciences, Beijing, China; 10 Department of Psychiatry and Human Behavior, University of Mississippi Medical Center, Jackson, MS USA; 11 Department of Pathology, Aarhus University Hospital, Aarhus, Denmark

**Keywords:** Molecular neuroscience, Scientific community

## Abstract

Brodmann Area 46 (BA46) has long been regarded as a hotspot of disease pathology in individuals with schizophrenia (SCH) and major depressive disorder (MDD). Pyramidal neurons in layer III of the Brodmann Area 46 (BA46) project to other cortical regions and play a fundamental role in corticocortical and thalamocortical circuits. The AutoCUTS-LM pipeline was used to study the 3-dimensional structural morphology and spatial organization of pyramidal cells. Using quantitative light microscopy, we used stereology to calculate the entire volume of layer III in BA46 and the total number and density of pyramidal cells. Volume tensors estimated by the planar rotator quantified the volume, shape, and nucleus displacement of pyramidal cells. All of these assessments were carried out in four groups of subjects: controls (C, *n* = 10), SCH (*n* = 10), MDD (*n* = 8), and suicide subjects with a history of depression (SU, *n* = 11). SCH subjects had a significantly lower somal volume, total number, and density of pyramidal neurons when compared to C and tended to show a volume reduction in layer III of BA46. When comparing MDD subjects with C, the measured parameters were inclined to follow SCH, although there was only a significant reduction in pyramidal total cell number. While no morphometric differences were observed between SU and MDD, SU had a significantly higher total number of pyramidal cells and nucleus displacement than SCH. Finally, no differences in the spatial organization of pyramidal cells were found among groups. These results suggest that despite significant morphological alterations in layer III of BA46, which may impair prefrontal connections in people with SCH and MDD, the spatial organization of pyramidal cells remains the same across the four groups and suggests no defects in neuronal migration. The increased understanding of pyramidal cell biology may provide the cellular basis for symptoms and neuroimaging observations in SCH and MDD patients.

## Introduction

The dorsolateral prefrontal cortex (DLPFC) has long been associated with dysfunction in psychiatric disorders such as major depressive disorder (MDD) and schizophrenia (SCH). Given the similaritites in genetic risk factors and symptom patterns between SCH and MDD, it is possible that the occurrence of comorbid depression in SCH is caused by a common etiological mechanism [1, 2]. Subjects with SCH and MDD perform poorly on cognitive tasks that rely on the DLPFC circuitry, and this poor performance has been linked to abnormal activation of the neural network in this brain region [3–9]. Working memory depends on the stability and functionality of the DLPFC, but schizophrenic patients perform poorly on working memory tasks due to this decreased activation [10–13] In addition, the amount of gray matter volume in the DLPFC has been found to be reduced in SCH and MDD patients compared to C subjects [14–18], and DLPFC injuries was found to be related to the intensification of depressive symptoms [19]. Alternative treatment such as repeated transcranial magnetic stimulation has been authorized by the US Food and Drug Administration to treat adult MDD, who have not responded to traditional treatments by stimulating neurons in the DLPFC [20–23]. The DLPFC is part of the neuronal circuit of emotion, which is connected to the limbic system, and any DLPFC dysfunction in people with SCH and MDD appears to have a substantial impact on the pathogenesis of both disorders. According to neuronal morphology studies, pyramidal neurons in layer III primarily project to other cortical regions and offer horizontal excitatory connections within the DPFC. This is particularly essential for corticocortical and thalamocortical circuits, as axon projections from the mediodorsal thalamic nucleus send information to pyramidal cells in deep layer III [24–33]. Studies have also shown a reduction in the mean somal size of all neurons in layer III of the DLPFC and a significant reduction in the density and dendritic spines of pyramidal cells in the deep layer III in SCH. A decrease in somal size has also been proposed to impact afferent and/or efferent connections in SCH [30, 34] as the somal size corresponds with the amount of dendritic and axonal arborization in a cell [24, 35]. Thus, understanding the structural changes related to the illness process requires examining the cellular and circuitry abnormalities that result in abnormal DLPFC neuronal activity in MDD and SCH patients. It is worth mentioning that suicidal thoughts are more prevalent in people with SCH and MDD, and both illnesses increase the chance of committing suicide [36–41]. Suicide victims with depression are typically included in postmortem research for MDD [42, 43] despite the fact that suicide has been discussed as having its unique neurobiology [44]. For instance, it is well understood that the insula is linked to the neural network associated with psychological pain and is anatomically significant in patients who have committed suicide [45, 46]. Insula is densely packed with von Economo neurons, which bear receptors for neurotransmitters that help in the regulation of complex emotions like empathy, remorse, and shame, and their density is significantly increased in suicide victims [47]. Consequently, the outcome of post mortem research may be affected, if suicidal characteristics specific to subjects who committed suicide (SU), rather than those who had MDD, are not differentiated [48, 49]. However, it is unclear whether different biological processes cause distinct structural and morphological changes in the prefrontal cortex between subjects with depression who committed suicide and subjects with depression who did not commit suicide.

The aim of this research is to investigate if MDD, SCH, or SU are associated with a decrease in the volume of layer III in the DLPFC of Brodmann Area 46 (BA46) and/or cause alterations in the number, density and morphological characteristics of pyramidal cells. To examine the differences between groups, we employed the most recent 3-dimensional (3D) tissue reconstruction and cellular analysis from a biopsy of BA46 to assess the volume, sphericity, orientation, and diameter of pyramidal cells and their spatial organization in layer III of archived human brain tissue. In addition to acquiring 3D structural data and organization of pyramidal cells from the biopsies, we used stereology to estimate the total volume, total pyramidal cell number, and density of layer III in BA46. Volume tensors obtained by the planar rotator quantified the average volume, shape, and orientation of pyramidal cells in layer III in BA46.

## Materials and methods

### Subjects

This study comprised deceased subjects with a documented psychiatric disorder assigned to a medico-legal autopsy in Denmark between 1947 and 2000. A total of 39 formalin-fixed human postmortem brains were obtained from two Danish brain banks and split into four subgroups of 10 unaffected control subjects (C), 10 subjects with schizophrenia (SCH), 11 subjects who committed suicide with a history of depression (SU) and 8 subjects with major depressive disorder (MDD) without committing suicide. The individuals were all Scandinavian Caucasians with no history of alcohol and drug misuse, and were collected in accordance with Danish law and with the consent of the Central Denmark Area Health Research Ethics Committees (license number: M-2017-91-17). A group of skilled psychiatrists diagnosed the MDD and SCH subjects in the study during previous and extended hospital admissions. Two very experienced psychiatrists (AB Bertelsen and R Rosenberg) reassessed and verified these diagnoses by carefully reviewing all medical records and comparing the diagnoses with the modern criteria of DSM-IV (Diagnostic and Statistical Manual of Mental Disorders, 4th Edition) and ICD-10 criteria (The International Statistical Classification of Diseases and Related Health Problems 10th Edition). A detailed list of information of each subject can be found in Supplementary Note 1, see Table S1.

### Sampling of BA46

A whole hemisphere was prepared for each patient by an experimenter (NYL) blinded to different groups, and BA46 was identified at the macroscopic level using the most recent atlas of its location by Grazyna Rajkowska [50]. Then, a tissue block comprising the whole BA46 was extracted and prepared for processing from the hemisphere, see Fig. S1. Subsequently, one biopsy of 3 mm in diameter was randomly chosen from a user-defined region based on the MATLAB script from GitHub [51]. The biopsy sample was chosen on the crown of the gyrus rather than near the sulci to ensure that the biopsy was perpendicular to the pial surface of the brain. The biopsies of BA46 were employed to assess the organization and morphological properties of pyramidal cells in layer III using 3D images. In the remaining tissue blocks containing BA46, stereology was used in layer III to statistically estimate volume tensors up to the second rank of pyramidal cells from a single optical plane to obtain information about the size, shape, and orientation of the cells to approximate the cell population and to detect morphological differences.

### 3D-analysis sample preparation

**Fig. 1:**
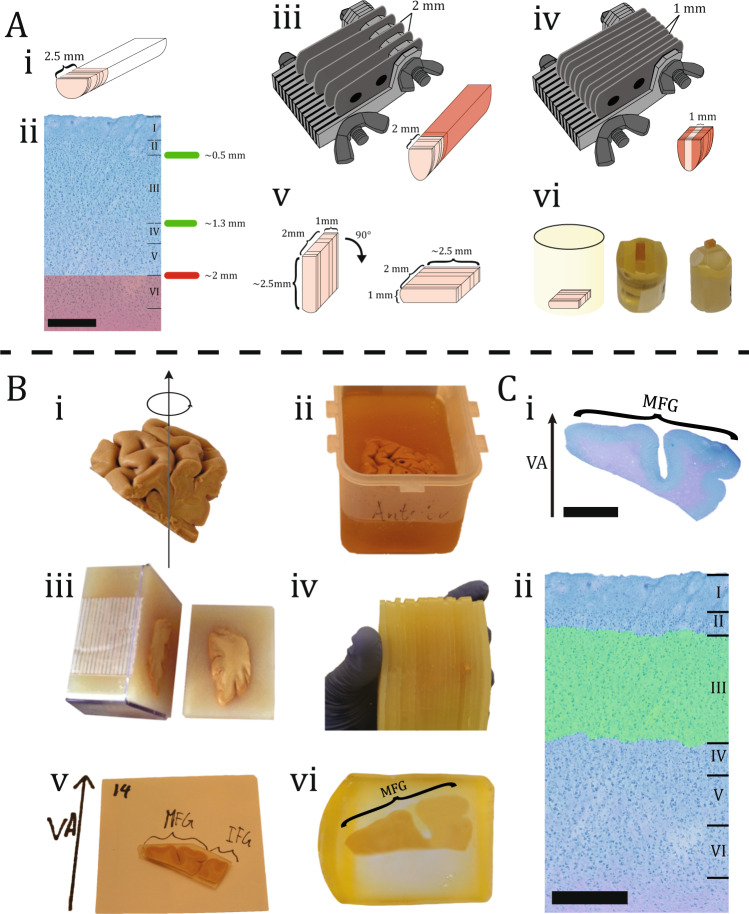
Sample preparation. **A** Biopsy preparation of the laminar site of the biopsy from BA46 is depicted in this illustration, with an average height of 2.5 mm (i). The six layers of BA46 can be seen in a cross-section of a biopsy after it has been stained with toluidine blue. Scale bar = 500 µm. Layer III is located within 2 mm of the pial surface (ii). A razor knife with a 2 mm gap between each blade was flipped on top of the biopsy. The red area shows the removed tissue (iii). A razor knife with a 1 mm gap between each blade was flipped on top of the biopsy. The red area shows the removed tissue (iv). The dimension of the tissue can be seen after the excess tissue has been removed. Before inserting the tissue within the embedding mold, it was flipped 90^◦^ to make sure the layers were parallel to the cutting direction (v). The illustration shows the tissue in the embedding mold. The resin block was photographed after it was trimmed with a glass knife, so it only contained layers I–IV (vi). **B** Tissue block preparation of DLPFC containing BA46 was rotated uniformly around a vertical axis (VA), perpendicular to the block’s central pial surface (i). The tissue block was embedded in a box filled with 7% agarose (ii). Next, the tissue block was cut systematically into uniformly random into 2.5 mm slabs orthogonal to the anterior-posterior direction (iii). The tissue block was then reassembled and every second the slab was systematically sampled (iv). After that, each slab was documented and various gyri positions were specified, e.g., medial frontal gyrus (MFG), inferior frontal gyrus (IFG) (v). Finally, the slabs were embedded in glycolmethacrylate (vi). **C** The section were stained with toluidine blue and VA was positioned based on documentation. Scale bar = 6 mm (i). The stereological analysis was then performed in layer III of BA46 based on the cytoarchitecture. Scale bar = 500 µm (ii).

The neocortical layers were parallel to the cutting direction and visible during the tissue sample because the biopsies were extracted perpendicular to the pial surface. The whole procedure is visualized in Fig. 1A. The gray matter in BA46 has a thickness of about 2.5 mm and covers all six neocortical layers. All additional tissues beyond 2 mm were removed from the sample with a razor knife, since layer III is the focus of this study and is definitely within the first 2 mm. Furthermore, the biopsies were then cut with a razor knife to a depth (*z*-height) of 1 mm and subsequently embedded in resin. After the resin had cured entirely, the sample was cut with a glass knife such that it only contained neurons in layers I–IV (see Supplementary Note 2 for additional details).

### 3D-analysis section collection and imaging

The resin-embedded biopsies were cut into serial sections using the Automatic Collector of Ultrathin Sections for Light microscopy (AutoCUTSLM), an ultramicrotome (Leica EM UC7) connected to a custom tape collection device. The serial sections for each subject were cut at a thickness of 300 nm and dyed with 1% toluidine blue before being mounted to glass slides.

The images of the sections were acquired using a Leica Apiro Versa 200 Digital Pathology Scanner with a pixel sample size of 272 nm (lens 20x, NA 0.8). Because the sections were 300 nm thick, it was decided to systematically sample every third section, resulting in ~1000 images with 900 nm sections. Images were saved for each section using MATLAB scripts released on GitHub [51], and the procedure is detailed in a previous study [52].

### 3D-analysis image processing

**Fig. 2:**
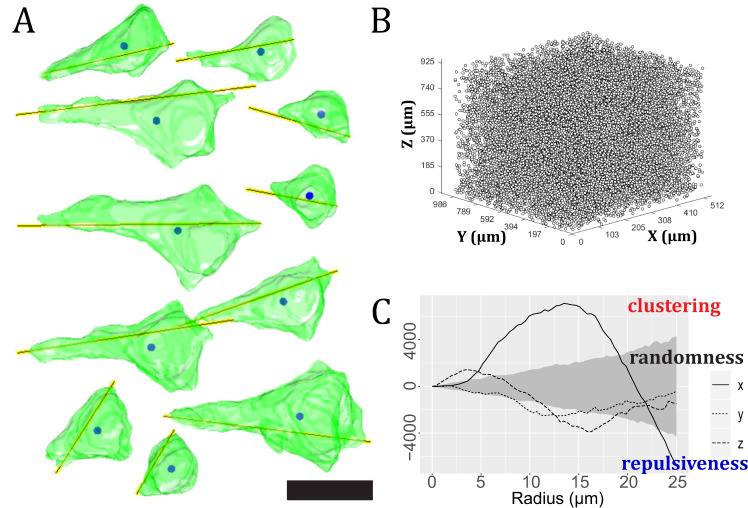
Quantitative measurements of 3D-reconstruction. **A** Pyramidal cell reconstruction in 3D, with yellow lines denoting orientation and solid circles denotes the centroid of pyramidal cells. Scale bar = 20 µm. **B** Point pattern consisting of centroids of pyramidal cells in layer III of BA46 acquired from a biopsy. **C** The results of a spatial point pattern analysis of pyramidal cell centroids using the cylindrical *K*-function. The empirical cylindrical *K*-function in the directions of the three axes is shown as three curves: *x*-axis (solid line), *y*-axis (dotted line), or *z*-axis (dashed line). The observed point pattern is completely random only if the curves fall within the envelope (gray area). When a curve falls above the envelope, it indicates evidence of clustering; when a curve falls below the envelope, it indicates evidence of repulsiveness. If the curve in one direction is particularly high, it indicates cylinder-shaped clusters in this direction.

The individual image sections for each subject had to be aligned before 3D data analysis could be performed. Therefore, a sequential section-to-section imagebased registration approach was employed to align the segmented sections. The pyramidal cells in layer III of BA46 were reconstructed in 3D to assess their morphological characteristics and spatial point pattern in 3D space. We used the Convolution Neural network UNetDense architecture to detect pyramidal cells [53]. Layer III was identified using a customized MATLAB algorithm, and quantitative data like volume, diameter, sphericity, and orientation were estimated for each pyramidal cell. Furthermore, the estimated centroids for each 3D-reconstructed pyramidal cell were used as coordinate points to study the spatial point pattern in layer III of BA46. The cylindrical *K*-function was employed to detect columnar patterns in the 3D point patterns for each subject and was used to make statements about clustered, random, and repulsive behavior along the three major axes (x, y, z) as previously described in detail [52], see Fig. 2.

### Stereology block preparation

After removing the tissue block containing BA46 from the hemisphere, vertical uniform random sections were applied to the tissue with a vertical axis (VA) chosen to be perpendicular to the pial surface, see Fig. 1B. In the bottom of the box, a paper ruler was placed, and the tissue block was rotated around the VA before being embedded in 7% agarose. After the hardening of the agarose, the block was cut into uniform random 2.5 mm thick parallel vertical slabs perpendicular to the anterior-posterior position using the Systematic Uniform Random Sampling procedure. The VA and neuroanatomy markers of each slab were then delineated, numbered, and documented. Following that, each slab was subsequently embedded in glycolmethacrylate (Technovit 7100) and cut into 40 µm sections, dyed with a Toluidinblue-Borax Solution and mounted with Eukitt.

### Stereology analysis

Prior to stereological analysis, each section was scanned using an Olympus scanning microscopy with an Olympus 20x oil lens. OlyVIA v.3.2 (Olympus, USA) is a high-resolution image viewer program that allows any user to zoom in and out of the section, making it easier to delineate BA46 from other nearby brain regions based on cytoarchitecture. The computer was then configured with the newCast software package VIS version 2017.7.1.3832 (Visiopharm, Hørsholm, Denmark), which enables stereological probes (points and counting frames) to be superimposed on histological sections. The amount of field of view (FOV) needed to investigate each sector was established via a cost-effective analysis based on pilot counts; thus, about 200–400 points were counted for each subject (see specifications in Table S2). To avoid bias, all histology sections were blinded by the investigator (NYL) to specific diagnostic cases while counting.

### Stereology—calculation of volume, number, density, and volume tensors

To estimate the volume of layer III in BA46, the Cavalieri estimator was combined with a point-counting stereology method. The volume fraction was estimated using Eq. 1 and all points within the specified ROI (layer III) were consistently counted, see Fig. S2A.

1 $$V = {{T}}\frac{a}{p}{\sum} P$$

where *V* is the volume, *T* is the mean distance between slabs, *a/p* is the area per point, and *P* is the total number of counted points. The optical fractionator probe was used to determine the total number and density of pyramidal cells in layer III of BA46 [54]. Pyramidal cells were consistently counted when the nucleolus was in focus inside an unbiased counting frame. Then, the total number of pyramidal cells, *N*, was calculated using the following formula:

2 $$N = \frac{1}{{ssf}}\frac{1}{{bsf}}\frac{1}{{asf}}\frac{1}{{hsf}}{\sum} {Q^ - } = 2\frac{T}{{MA}}\frac{A}{a}\frac{{t_{Q^ - }}}{h}{\sum} {Q^ - }$$

where 1*/ssf* is the section sampling fraction and is equal to 2 (every second slab was sampled), *bsf* the block sampling fraction, *asf* the area sampling fraction, *hsf* the height sampling fraction, *Q*^*−*^ the total number of sampled pyramidal cells in layer III, *T* the mean distance between slabs, *MA* the microtome advance (section thickness), *A* the area of the sampling grid, *a* the area of the counting frame, *h* the disector height and *t*_*Q*_^*−*^ the number-weighted mean section thickness. The section thickness was 40 µm, and because the sections were embedded in glycolmethacrylate, the shrinkage was minimal, albeit the average section thickness, *t*_*Q*_^*−*^, was about 36 µm. As a result, the counting frame for the *z*-axis analysis was set at the height of 20 µm after a *z*-axis analysis, see Fig. S2B.

The planar rotator probe has previously proven to be able to calculate volume tensors and was utilized in this study [55]. The sample area was selected perpendicular to the specified VA from the central gyral region of BA46 for each subject. The reference point was the nucleolus of a neuron, and four half-lines were employed, see Fig. S3. The volume tensor of rank *k* associated with a pyramidal cell *X* is given by

3 $$T_k\left( X \right) = \frac{1}{{k!}}{\int}_X {x^kdx}$$

where *x*^*k*^ is the symmetric tensor of rank *k*, as determined by *x* = (*x*_1_*, x*_2_*, x*_3_) ∈ R^3^ and the integration is done with respect to the volume calculation in R^3^.

It is then possible to obtain information about the volume, shape, and orientation of *X* by combining volume tensors of rank 0, 1, and 2 [55].

### Statistical procedures

We performed a one-way analysis of variance (ANOVA) with a 95% confidence interval to assess differences between the four groups. In the multiple comparisons test, a Bonferroni correction (post hoc test) was performed to avoid type 1 errors. We employed random permutations to build the 95% global envelope for testing whether the cylindrical *K*-function differed between the four groups. The null hypothesis states no differences in the cylindrical *K*-function across groups, and it cannot be rejected if and only if the observed curve falls inside the envelope. If the curve falls outside the envelope at any point, it thus means that there are significant differences in the cylindrical *K*-function. The test is described in detail in Supplementary Note 3.

## Results

### Morphological 3D data

**Fig. 3:**
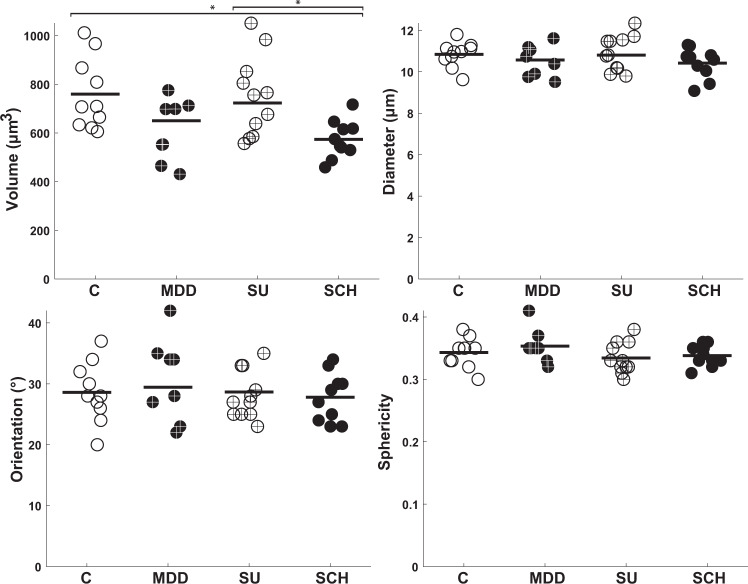
Morphological results of the 3D-analysis of BA46 in layer III. The estimated volume, diameter, orientation, and sphericity of pyramidal cells in layer III of BA46 between the four groups. Control (C), Major depressive disorder (MDD), Suicide (SU), and Schizophrenia (SCH). ^∗^*P* < 0.05, ^∗∗^*P* < 0.01, ^∗∗∗^*P* < 0.001.

Figure 3 and Tables S3–S6 present the descriptive statistics for the volume, diameter, orientation, and sphericity of the 3D-reconstructed pyramidal cells in layer III of BA46. The volume of pyramidal cells was significantly different among the four groups (*F* (3,35) = 4.96, *p* = 0.0057). The volume of pyramidal cells was significantly smaller in SCH compared to C and SU with *p*-values of 0.02 and 0.03, respectively. On the other hand, the diameter, orientation, and sphericity did not significantly differ across the groups, since (*F* (3,35) = 1.09, *p* = 0.37), (*F* (3,35) = 0.75, *p* = 0.53), and (*F* (3,35) = 1.16, *p* = 0.34), respectively.

### Spatial point pattern analysis

**Fig. 4:**
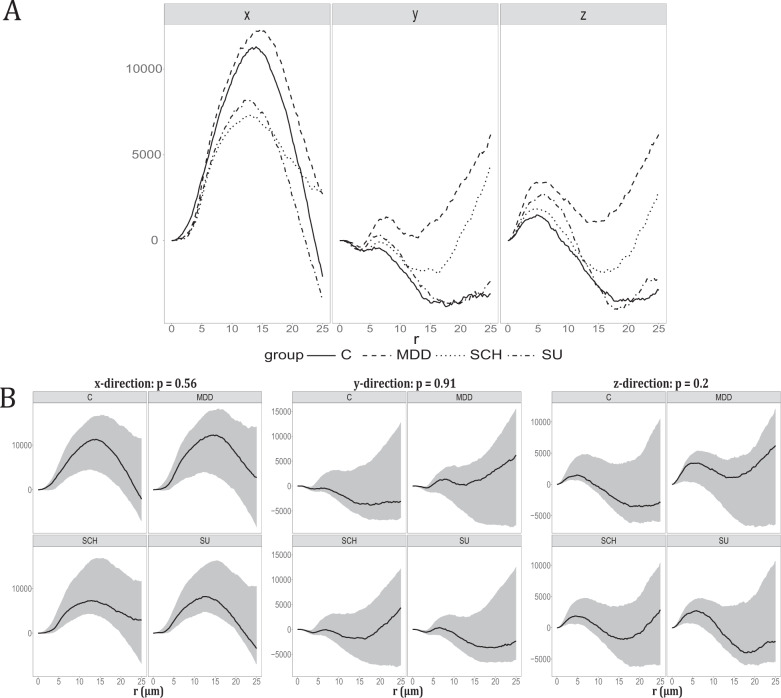
Spatial point pattern analysis. **A** Plot of the weighted mean of each group. The weighted mean of the empirical cylindrical *K*-functions minus the theoretical value under CSR in the direction shown at the top. The line type corresponds to the groups: Control (C), Major depressive disorder (MDD), Suicide (SU), and Schizophrenia (SCH) as stated in the legend. **B** Test the difference between groups in the *x*, *y*, and *z*-axes.95% Global envelope test for testing the hypothesis of no differences in the cylindrical K-functions of the four groups Control (C), Major depressive disorder (MDD), Suicide (SU), and Schizophrenia (SCH). There is a test for the cylindrical *K*-function in each of the directions of the *x*-, *y*-, and *z*-axes. The 95% global envelopes (gray area) were based on 8000 random permutations. The solid curves are the estimates obtained from data after the theoretical value under CSR was subtracted. The direction is stated at the top of each plot, as well as the *p*-value of the test.

The spatial organisation of centroids was examined for each subject by estimating the cylindrical *K*-function from their 3D point patterns. Based on the cylindrical *K*-function, we found clear evidence of columnar clusters in the direction of the *x*-axis, which is perpendicular to the pial surface, for all 10 subjects in C, 7 in MDD, 7 in SCH, and 3 in SU, see Figs. S6–S9 in Supplementary Note 3. We visualized the overall behavior of the empirical cylindrical *K*-function in the *x*, *y*, and *z*-directions within each group by plotting a weighted mean, see Fig. 4A. Figure 4A also clearly shows that there is an overall tendency for columnar clusters in the direction of the *x*-axis in all four groups. In 16 subjects, the cylindrical *K*-function also detected some repulsive behavior between cell centroids.

The global envelope test found no significant differences of the cylindrical *K*-function between the four groups (Fig. 4B). Additional tests also found no differences in Ripley’s *K*-function, the empty-space function *F*, and the nearest-neighbor function *G*. Supplementary Note 3 includes a description of the entire point pattern analysis, including specific figures for each subject, and results of the additional tests.

### Stereology and volume tensors

**Fig. 5:**
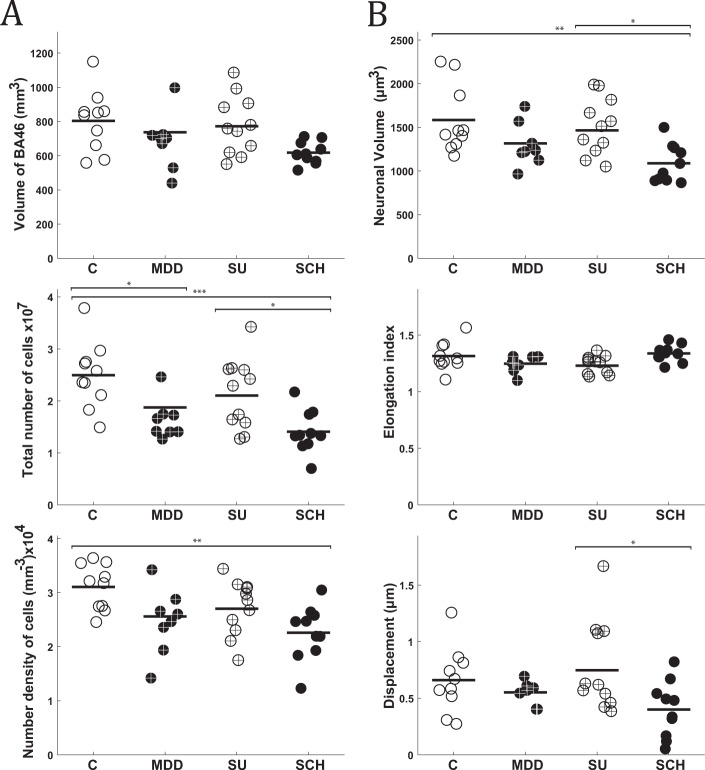
Morphological results of the stereological analysis of BA46 in layer III. **A** Stereological measurements of the total volume, total neuronal number of pyramidal cells, and number density of pyramidal cells in layer III of BA46. **B** Volume tensor estimates of pyramidal cell somal size and shape in layer III of BA46, including volume, elongation index, and displacement. Control (C), Major depressive disorder (MDD), Suicide (SU), and Schizophrenia (SCH). ^∗^*P* < 0.05, ^∗∗^*P* < 0.01, ^∗∗∗^*P* < 0.001.

Tables S7–S12 in Supplementary Note 2 provides the descriptive statistics for the stereological measures in layer III of BA46 (volume, neuronal number, neuronal density, neuronal volume, elongation index, and displacement) among the four groups, and Fig. 5 shows a visual depiction of the data. Five of the descriptive statistics revealed a statistically significant difference in the one-way ANOVA: BA46 volume (*F*(3,35) = 3.22, *p* = 0.034), neuronal number (*F*(3,35) = 7.69, *p* = 0.0004), neuronal density (*F*(3,35) = 5.06, *p* = 0.005), neuronal volume (*F*(3,35) = 4.50, *p* = 0.009), and displacement (*F*(3,35) = 3.34, *p* = 0.033). There was no statistically significant difference in comparing the volume of BA46 between individual groups, despite a borderline significant difference between C and SCH with a *p*-value of 0.056. The total number of pyramidal cells in SCH and MDD were significantly different from C, and the difference between SCH and SU was also significant, with *p*-values of 0.0014, 0.0006, and 0.029, respectively. The number density of pyramidal cells was significantly smaller in subjects with SCH compared to C with *p* = 0.004.

The volume of pyramidal cells in patients with SCH was smaller compared to C and SU, as was also found with the 3D-reconstruction measurements, with *p*-values of 0.009 and 0.04, respectively. There was a significant difference in the nucleus displacement between the SU and SCH, with a *p*-value of 0.03.

## Discussion

Our findings showed that subjects with SCH had a 23% decrease in the estimated layer III brain volume of BA46 compared to C, and this correlated with a decrease in the total number, density, and somal volume of pyramidal cells. Measured attributes from MDD subjects tended to follow SCH, but there was only a significant decrease in pyramidal total cell number compared to C. Although we found no differences in any of the morphometric measurements between SU and MDD, SU had a significantly higher total number of pyramidal cells and nucleus displacement than SCH.

The 3D spatial organization of pyramidal cells in BA46 revealed that they were arranged into smaller columnar structures or displayed repulsive behavior, and when the four groups were compared, no statistically significant difference was found. This implies that there are no abnormalities in neuronal migration, where neurons are traveling through the coloumnar structure, suggesting that SCH, MDD, and SU are not neuronal migration disorders.

### Morphological 3D data

We found a significant reduction in the neuronal volume of pyramidal cells in SCH compared to C and SU subjects, approximately 24% and 23%, respectively. Other independent studies have documented a reduced volume of pyramidal cells in layer III in the prefrontal cortex of SCH patients using different sampling and volume estimation of cells, which is consistent with our findings [30, 34, 56, 57]. The lower soma volume is consistent with other abnormalities observed in schizophrenia patients, such as decreased dendritic spine density and total length of pyramidal cell dendrites in layer III of the DLPFC [58–61] Given the role of pyramidal cells in layer III in connection with corticocortical and thalamocortical interactions, the amount of cortical and thalamic excitatory inputs is likely to have changed [62]. The 3D reconstruction of pyramidal cells in layer III found a significantly lower volume for the SCH subjects when compared to SU, whereas the volume in subjects C and SU was almost the same and the *p*-value was 1. In agreement with our results, a recent postmortem study found no significant differences in neuronal volume, neuronal density, or glial density between unaffected controls, suicidal individuals with early-life adversity, and suicidal subjects without early-life adversity [63]. Suicidal behavior is thus proposed to be addressed as a separate illness in an analysis of data from neurochemical and genetic tests that demonstrate decreased levels of the neurotransmitter serotonin, neurotransmitter receptors, and structural changes in the prefrontal cortex [44, 64, 65]. There were no significant differences in orientation or sphericity measures across the four groups. As a result, we may conclude that the apical dendrites of pyramidal cells do not endure orientation changes and are unaffected by MDD, SU, and/or schizophrenia. If the neuronal volume is altered, the shape cannot be expected to remain constant. The sphericity was used to assess the shape measurements of pyramidal cells and the shape does not change even when the volume decreases due to SCH, suggesting the volume loss is homogeneous.

### Spatial point pattern analysis

The cylindrical *K*-function was used to analyze the neural organization of centroids of pyramidal cells in layer III of BA46. We discovered significant evidence of a columnar structure in the direction of the *x*-axis in 27 subjects, especially when we looked at cylinders with radii ranging from 5 to 20 µm. As a result, smaller columnar formations perpendicular to the pial surface may represent the smallest functional unit of the mammalian brain’s cerebral cortex. Human minicolumns have previously been measured to have radii ranging from 17.5 to 30 µm [66–70]. However, the fact that the neural organization in 3D space was evaluated using 2D techniques may have resulted in misrepresenting biological structures and cell malpositioning in 3D space. Furthermore, while those 2D approaches rely on a priori models that assume a minicolumnar structure, our methodology does not. Moreover, the 2D procedures used to investigate minicolumns were used in different locations compared to BA46. Aside from practicing different methodologies, the variation in minicolumn size can also be explained by differences in number and volume of neurons in multiple brain regions [71, 72]. Hence, columnar size is unlikely to be constant across all brain areas. We were unable to identify any significant differences among the four groups of the cylindrical *K*-function using the global envelope test.

### Volume and number of cells in layer III of BA46

In layer III of BA46, stereology and volume tensors were used to measure total volume, number of pyramidal cells, neuronal density, and neuronal characteristics such as neuronal volume, elongation index, and displacement. In our study, the ANOVA detected a statistical difference between the four groups in the volume of layer III in BA46. However, the post hoc test could not detect any significant differences between the groups despite a strong tendency toward smaller volume of SCH than controls, as demonstrated by the *p*-value of 0.056 and a 23% difference. In patients with schizophrenia, multiple neuroimaging and post mortem studies have revealed a decreased gray matter volume in the prefrontal cortex. However, BA46 does not fill the entire frontal lobe, and we only measured the volume of layer III, not all six layers.

In SCH and MDD, the total number of pyramidal cells differed substantially from C, as well as between SCH and SU subjects. Given that layer III of BA46 is closely connected to the corticocortical and thalamocortical circuits, a reduction in pyramidal cell number may affect neuronal connectivity. The notion of altered connectivity in the prefrontal cortex is supported by recent neuroimaging studies of impaired white matter connections in patients with SCH and MDD [73–76]. Other studies have also revealed a loss in neuronal numbers in the prefrontal cortex and other brain regions of SCH patients, including the anterior cingulate region, primary visual cortex, primary motor cortex, hippocampus, mediodorsal thalamic nucleus, and thalamus [77–82]. Other studies have found no evidence of neuronal cell death in the prefrontal cortex and hippocampus [83–86]. Hence, there is no consensus in the literature on whether there is evidence for neuronal loss in the brains of individuals with schizophrenia. Given the heterogeneity of schizophrenia, a complex process of neuronal cell death may occur to varied degrees in different people and brain areas. To the best of our knowledge, no studies on the total number of neurons in the prefrontal cortex or BA46 in MDD or SU subjects have been performed. Nonetheless, certain studies have employed stereology to assess neuronal and glial cell density in the cortex, where a reduction in somal size, neuronal and glial density has been reported in the rostral orbitofrontal region [87]. In contrast, most MDD studies did not find any difference in neuronal density, which agrees with our findings [88–91] When using densities to make any definitive statements about changes in 3D structures, it is important to realize that cell number density is only a set of ratios and do not reflect changes in the total number of cells. Because the density can rise if the number of cells remains constant but the volume of the examined region decreases. Although SU has also experienced depression, SU resembles C more than MDD, which could explain the difference in neuronal number between SU and SCH. This may be due to the fact that SU subjects did not fully develop MDD. Another possibility is that SU might be considered a separate disorder. Growing evidence suggests that depression increases the risk of neurodegenerative illnesses, including Alzheimer’s, Parkinson’s, and Huntington’s disease [92, 93]. It thus has a substantial influence on the growing burden of these neuropathologies.

Regarding neuronal density, the difference between C and SCH was significant. The neuronal density of SCH patients was 27% smaller than controls in our study, which agrees with a prior finding in the prefrontal cortex [77, 94]. However, the differences in neuronal density are subject to disagreement because other studies have reported neuronal density to be greater in SCH patients [95, 96]. Technical considerations, such as the tissue embedding media, could also have an impact on density estimates. Furthermore, since the density estimates are based on five evenly spaced section measurements, in these studies, the total volume or reference volume of the measured BA46 was not defined. For example, suppose the total neuronal number remains constant while the reference volume decreases but is not taken into consideration. In this case, the density measurement may be biased, resulting in a higher-than-expected density estimate. This is especially problematic in SCH patients, who have a decreased amount of neuropil in the cortex caused by the condition.

### Volume tensors

We detected a decrease in the neuronal volume of pyramidal cells in layer III of BA46 in SCH compared to control and suicidal subjects, similar to the results obtained from the 3D-reconstruction dataset. The volume differences for SCH compared to C and SU subjects are about 28% and 24%, implying a comparable volume loss to the 3D-reconstruction, which showed a difference of 24% and 23%, respectively. The estimated elongation index of pyramidal cells in layer III of BA46 showed no significant difference between the four groups, which matches our findings regarding the shape measurement sphericity in the 3D-reconstruction. Thus, although the neuronal volume changes significantly, the shape of a neuron remains unchanged. The shape cannot be expected to remain constant because neuronal volume varies significantly between subjects. Even if two objects or cells have the same volume, their shape can be quite different. Our findings show that the shape of pyramidal cells are unaffected, meaning that the volume decrease occurs uniformly. As a result, the assumption of isotropy for these human pyramidal cells is not entirely incorrect. In addition to calculating the elongation index of pyramidal cells, the volume tensor calculations provided information on the displacement, which is the distance between the location of the nucleolus (reference point) and the approximate center of mass in our samples. Our findings revealed a significant difference in the nucleus displacement between SU and SCH, with distances of 0.40 µm and 0.78 µm, respectively, showing that the nucleus of an SCH subject is closer to the center of mass.

The nucleolus participates in protein synthesis, transcription, and processing of ribosomal RNA (rRNA) in cells [97]. Environmental stimuli like stress are known to feed into the tight management of rRNA production, making the nucleolus the central focus of the cellular stress response. One of the methods which cells use to preserve cellular energy balance under stressful situations is to reduce ribosome biosynthesis [98, 99]. Cellular responses such as activation of cell repair mechanisms, autophagy, or cell death vary depending on the stressor. Assume that the cells have been exposed to stress-induced internal and external stimuli over a long period of time or have gone through apoptotic events. If so, it may explain the differences in the nucleus displacement and the total number and volume of pyramidal cells in SCH subjects.

### Limitations of the approach or study

We were able to obtain an equitable balance of male/female and right/left hemisphere of BA46 in the current material, but the small number of subjects constrains the current study.

Brain specimens from two different brain banks were examined: The tissue for C and SU-groups was provided by Tissue Bank (Forskningsvævsbanken) at Core Center for Molecular Morphology, Section for Stereology and Microscopy, Department of Clinical Medicine, Aarhus University. Tissue from the MDD and SCH groups was obtained from Brain Collection at Translational Neuropsychiatry Unit, Aarhus University.

Despite the fact that the PMI of the two tissue collections differed, Nissl staining was unlikely to reveal a difference in the number of visible cells when all cells appeared to be stained. Hence, our cell number estimation should be resistant to this limitation. Nonetheless, PMI could cause potential changes in the observed volume of BA46. However, the observed mean cell volume was virtually similar across groups, except for SCH, which was lower and matched the literature, although with no significant differences. As a result, the volume changes seen are most likely biological, as evidenced by a within-group correlation analysis found no significant relationship between PMI and the volume of BA46 in layer III, total number of cells, cell density, or cell volume (see Fig. S4).

The subjects of C and SU lived and died in society, whereas MDD and SCH died in a psychiatric facility. Thus, the unknown effects of being admitted to a psychiatric facility cannot be overlooked. Living in a mental institution appeared to be beneficial for many subjects as they lived to old age. Because of the age imbalance between groups, age-related diseases such as arteriosclerosis and brain atrophy may influence our findings. However, no significant within-group correlation was found between age and the measured attributes, despite differences in mean subject age between the two brain collections (see Fig. S4).

Apart from the control group, the majority of the subjects have had medical therapy, and the effects of such therapies cannot be disregarded. Antipsychotic use in nonhuman primates has been linked to reduced brain volume and glial cell number, as confirmed by a significant human imaging study by Beng-Choon Ho et al., which included 211 people over a period of 7.2 years [100–103]. However, those studies found no differences in neuron counts. The fact that neither the MDD nor the SCH groups took drugs or consumed alcohol, nor did they commit suicide, is a strength of the study, considering patients in mental institutions have more severe disease manifestations than subjects, who live in society.

## Conclusion and perspectives

In conclusion, these findings demonstrate that the morphological alterations occurring in BA46 are consistent with the structural and neuro-plasticity theories of depression and schizophrenia.

In future postmortem human brain studies, it will be more beneficial to use a greater number of subjects to investigate the interplay between patient symptom profiles with clinical scores and treatment. The role of BA46 could benefit greatly by using the already prepared material to investigate glial cells and classify them into subtypes. Myelination has been shown to be reduced in SCH [104], oligodendrocytes are easy to distinguish, and they satellite around neurons to keep the proficiency elevated. Furthermore, the resin-embedded biopsies may also be viewed under an electron microscope to analyze cellular ultrastructure, and they could be used to bridge the localization of key molecules with light microscopy. The effect of these illnesses on other brain regions in the same material, such as the thalamus, insula, or other cortical areas, could also be studied using the same 3D-reconstruction procedure and stereology.

## Supplementary information

Supplemenraty information
