# Supplementary material for: Layer III pyramidal cells in the prefrontal cortex reveal morphological changes in subjects with depression, schizophrenia, and suicide
Source: Transl Psychiatry. 2022 Sep 5;12:363. doi: 10.1038/s41398-022-02128-0 (PMC9445178; doi:10.1038/s41398-022-02128-0)
Supplement: Supplementary file 1 — Supplemenraty information [file 41398_2022_2128_MOESM1_ESM.pdf]

Supplementary Note 1

Table S1: BA46 was taken from four groups of subjects (Control (C), Major depressive disorder (MDD), Suicide (SU), and Schizophrenia (SCH)) who were matched for sex, age, hand, post mortem interval (PMI), and storage time. More information can be found in the paper by Chen et al.[1], where the same subjects were studied.

#	Diagnosis	sex	Age (yrs)	Side△	PMI (hrs)	Storage time (yrs)	Cause of death†
**C**							
1	C	F	30.6	L	54	17.9	Pulmonary embolism
2	C	F	36.7	L	80	18.8	ASCVD
3	C	F	39.6	R	77	18.9	ASCVD
4	C[a]	F	53.6	R	24	19.9	ASCVD
5	C	F	65.5	R	30	17.0	ASCVD
6	C	M	26.8	R	40	19.7	Car accident
7	C	M	47.9	L	66	19.6	ASCVD
8	C	M	58.1	R	42	20.4	ASCVD
9	C[a,b]	M	69.9	L	106	19.6	ASCVD
10	Control	M	82.2	R	24	30.3	ASCVD
Mean		5F/5M	51.1	6R/4L	54.3	20.2	
CV*			0.35		0.51	0.18	
**SCH**							
1	SCH[c]	F	62.2	L		59.3	ASCVD
2	SCH	F	65.8	R	60	54.7	Pulmonary embolism
3	SCH	F	69.3	R	12	56.2	ASCVD
4	SCH	F	73.7	R	11	47.8	Kidney infection
5	SCH	F	81.0	L	43	49.5	Gastrointestinal infection
6	SCH	F	81.4	R	40	55.1	ASCVD
7	SCH	M	33.3	L	19	42.2	Respiratory failure
8	SCH[d]	M	60.3	L	2	60.2	Pulmonary embolism
9	SCH	M	78.9	R	31	56.6	ASCVD
10	SCH	M	79.9	R	17	47.6	Bronchopneumonia
Mean		6F/4M	68.6	6R/4L	26.1	52.9	
CV*			0.21		0.72	0.11	
**MDD**							
1	MDD	F	54.2	L	17	66.3	Bronchopneumonia
2	MDD	F	63.1	L	9	49.4	Pulmonary embolism
3	MDD	F	66.6	R	24	70.9	Bronchopneumonia
4	MDD[e]	F	77.5	L	24	48.0	Pneumonia
5	MDD	F	80.0	L		52.5	Pneumonia
6	MDD	F	81.8	R	25	41.7	Pulmonary embolism
7	MDD	M	32.6	R	24	67.9	Bronchopneumonia
8	MDD	M	73.9	R	54	47.6	ASCVD
Mean		6F/2M	66.2	4R/4L	25.3	55.5	
CV*			0.25		0.55	0.20	
**SU**							
1	SU[f]	F	28.7	L	38	17.8	Jumping
2	SU[g]	F	35.2	L	95	20.3	Suffocating
3	SU[h]	F	35.7	R	9	19.8	Drug overdose[l]
4	SU[f]	F	53.7	R	48	17.8	Knife lesion
5	SU[h]	F	69.2	R	30	20.3	Drowning
6	SU[h,i]	M	22.8	R	29	20.7	Suffocating
7	SU[f]	M	49.4	L	38	20.1	Hanging
8	SU[f]	M	54.1	R	160	20.4	Drowning
9	SU[j]	M	57.5	R	85	20.0	Knife lesion
10	SU[f,k]	M	67.1	L	41	20.4	Car exhaust poisoning
11	SU[g]	M	88.0	R	48	20.5	Hanging
Mean		5F/6M	51.3	7R/4L	56.5	19.8	
CV*			0.38		0.75	0.05	

[a] Total time in psychiatric care, including both psychiatric and somatic care due to psychiatric illness (time in parenthesis), [b] AD: Antidepressants, AP: Antipsychotics, APa: Anti Parkinson treatment, ECT: Electroconvulsive therapy, H: Hypnotics, ICT: Insulin coma therapy, S: Sedatives, [c] Number of single ECT treatments. [d] Number of single ICT treatments.

Supplementary Note 2

3D-analysis - sample preparation

The pyramidal cells in BA46 layer III were obtained from the extracted biopsies with a diameter of 3 *mm*. The direction of the biopsy should be such that the pial surface is perpendicular to the neocortex after sampling. Therefore, the neocortical layers were parallel to the cutting direction during tissue sampling and visible after staining.

The entire process is depicted in **Fig.1**. The grey matter in BA46 is approximately 2.5 *mm* thick and covers all six neocortical layers. All tissues beyond 2 *mm* were removed from the sample using a razor knife since only layer III is of interest in this study. The biopsies were then sliced with a razor knife to a z-height of 1 *mm* and immersed in Phosphate-buffered saline (pH 7.3) with sucrose for one day before being rinsed twice at room temperature for 5 minutes each time in 0.05 Mol maleate buffer (pH 5.2). The samples were then processed and embedded in a Leica EM TP Automated Tissue Processor (Leica Microsystems, Brønshøj, Denmark). The biopsies were dehydrated using a graded ethanol sequence after one hour of staining with 1% uranyl acetate in maleate solution (70%, 86%, 96% and 99%, 20 min each). After dehydration, the sample was washed three times in ten minutes with 100% acetone and then infiltrated with 100% acetone/epon 1:1 overnight for 12 hours with continuous rotation.
Infiltrated samples were incubated for one hour in pure Resin 812 before being placed in embedded molds and polymerized for 24 hours in a prewarmed oven (60°). The biopsies were positioned at the bottom of the embedded form with the pial surface perpendicular to the cutting direction of the knife. After the resin had cured completely, most of the white resin from the embedded sample was roughly trimmed using a high-speed milling machine (EM TRIM2, Leica) with an angle set at 60°. The first section of the sample was stained with 1% toluidine and inspected under a microscope to detect layer III. Next, a glass knife was used to trim approximately a $1{\times}1.4$ mm^2 area, leaving only neurons in layers I-IV in the sample.

Stereology - sample preparation

Figure **1B** portrays the tissue embedding procedure. The vertical axis (VA) was set to be perpendicular to the pial surface after the block of tissue containing BA46 was removed from the hemisphere. A paper ruler was inserted in the bottom of a box where the tissue block was mounted in 7% agarose. The block was sliced into uniform random 2.5 *mm* thick parallel vertical slabs

perpendicular to the anterior-posterior position using the SURS technique after the agarose had hardened. The tissue block was then reassembled, and every second the slab was systematically sampled. Anatomical landmarks and VA of each slab were then characterized and documented, including the superior frontal gyrus, medial frontal gyrus, and inferior frontal gyrus. The slabs were then numbered and placed in a tissue processing embedding cassette with foam pads to keep them safe while submerged in a solution. The tissue was then dehydrated in a Shandon Citadel 1000 tissue processor (Thermo Scientific, USA) using a 70%, 96%, and 99% ethanol series. Next, the tissue was submerged in glycol methacrylate for two shifts in 1:1 ethanol: Technovit 7100 solution, then in pure Technovit 7100 without the hardener for one shift. Each shift lasted one day in a 4° fridge. The tissue was then placed into an embedding mold after being withdrawn from the tissue processing embedding cassette. The tissue was put at the bottom of each embedding mold, which contained 15 ml of Technovit 7100. After that, 1 mL of hardener was applied, and the tissue took about five days to polymerize fully. The slabs were then cut into 40 μm sections, stained with 1% Toluidinblue-Borax Solution for 30 minutes, submerged in distilled water for 2 minutes, air-dried, mounted with Eukitt, and covered with 120 μm thick coverslips.

Stereological analysis

Every section was scanned using an Olympus scanning microscope with an Olympus 20x oil lens prior to stereological examination. The image viewer application OlyVIA v.3.2 (Olympus, USA) can display a high-resolution image with the option to zoom in and out of the slice, making it simpler to detect and deliniate BA46 based on the surrounding cytoarchitecture. The stereological study was then carried out for each subject in layer III of the whole BA46 using an Olympus BX51 light microscope with an Olympus DP70 camera (Olympus, Tokyo, Japan), an H138A motorized stage (Prior, Cambridge, UK) to load the microscopy slides, and stereological software VIS version 2017.7.1.3832 (Visiopharm, Hørsholm, Denmark). The amount of field of view (FOV) for each section was decided by the proportion of tissue that required to be examined to produce an accurate approximation, see **table S2**. This percentage was calculated by doing a cost-effective analysis based on pilot counts, with the objective of assessing an average of 2-300 points per participant. Layer III of BA46 was, therefore, ready to be sampled and examined using stereological probe sets in a systematic manner. Layer III of BA46 was then sampled and analyzed using stereological probes(Cavalieri estimator, optical fractionator probe, planar rotator). All

histological sections were counted while the investigator (NYL) was blinded to avoid bias.

Supplementary Figures

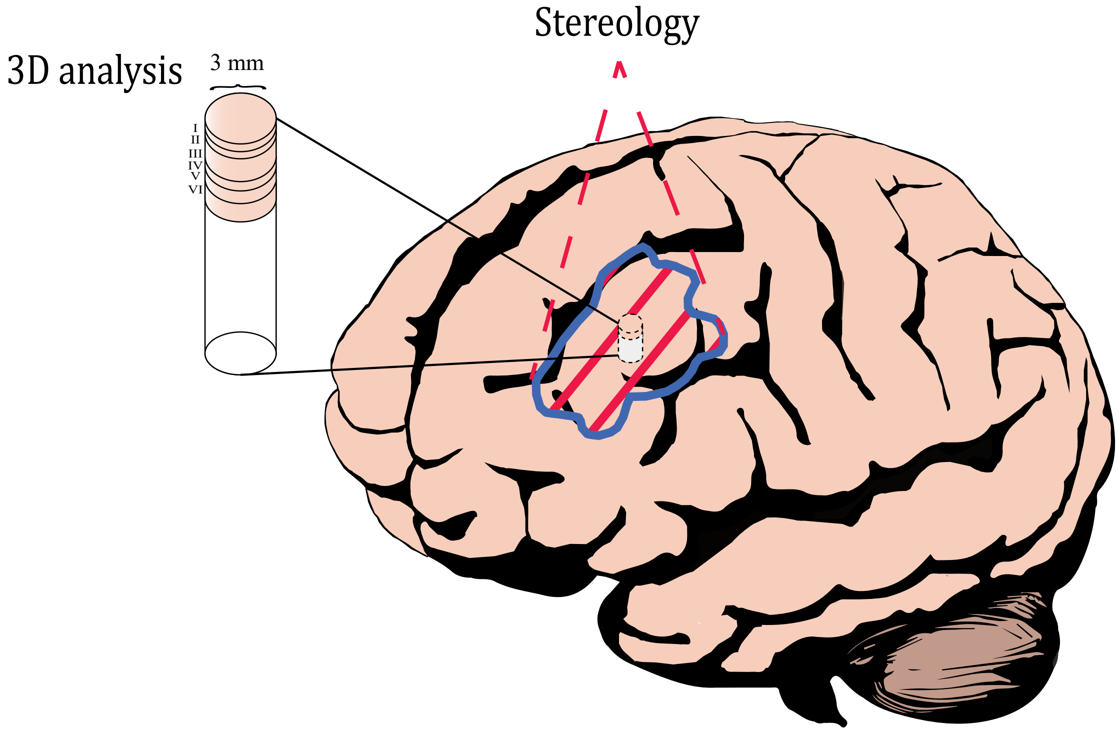

Figure S1: Biopsies were used for 3D analysis, while the remaining portion of the tissue containing BA46 was used for stereological analysis.

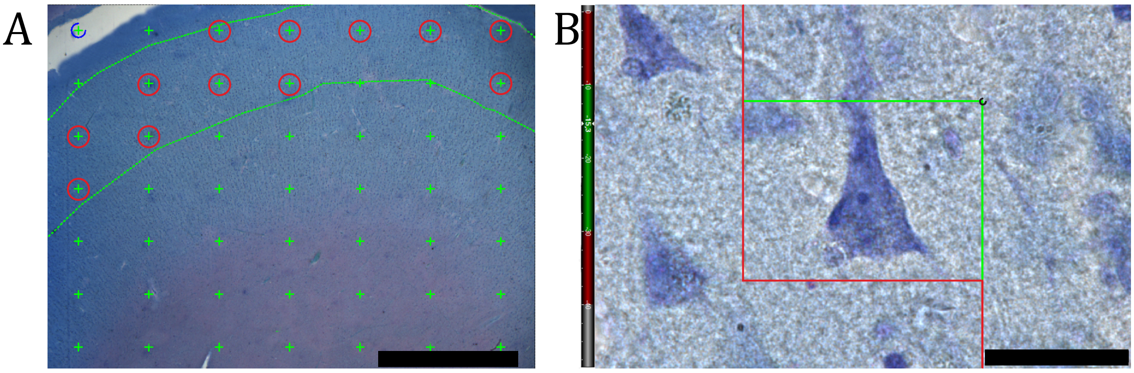

Figure S2: **(A)** A 7x7 point grid (green cross) was superimposed on the top of the section with a magnification of 1.25x. Layer III was within the region of interest (ROI) and was marked within green dashed lines. Points within the ROI were counted and represented with red circles. Scale bar= 2000 μm. **(B)** A field of view (FOV) with an x60 lens, which was superimposed on a counting frame (CF) on the top of the section. Only those cells that were within the box or touched the green line were counted. Cells that were outside of the box or hit the red line were excluded. Scale bar= 35 μm.

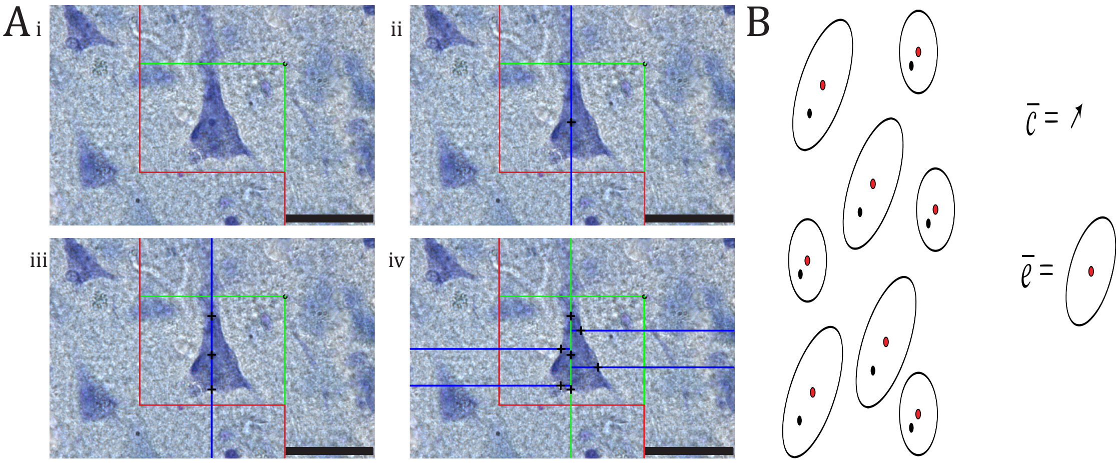

Figure S3: **(A)** Steps were used to sample a pyramidal cell with the Section Estimator. Within the optical disector, a neuron in focus (i). The blue line represents the predefined vertical axis, with the nucleolus serving as the reference point (ii). The top and bottom cell boundaries were marked (iii). There are four half-lines perpendicular to the vertical axis that appear in a uniform random position (iv). The + sign denotes the point where the neuron border and the half-line cross. Scale bar= 35 μm. **(B)** 2D representation of the displacement vector $\bar{c}$ from the nucleolus (reference point) to the center of mass and the Miles ellipsoid $\bar{e}$. $\bar{e}$ represents the population shape. A red circle represents a particle's center of mass, while a black circle represents the reference point.

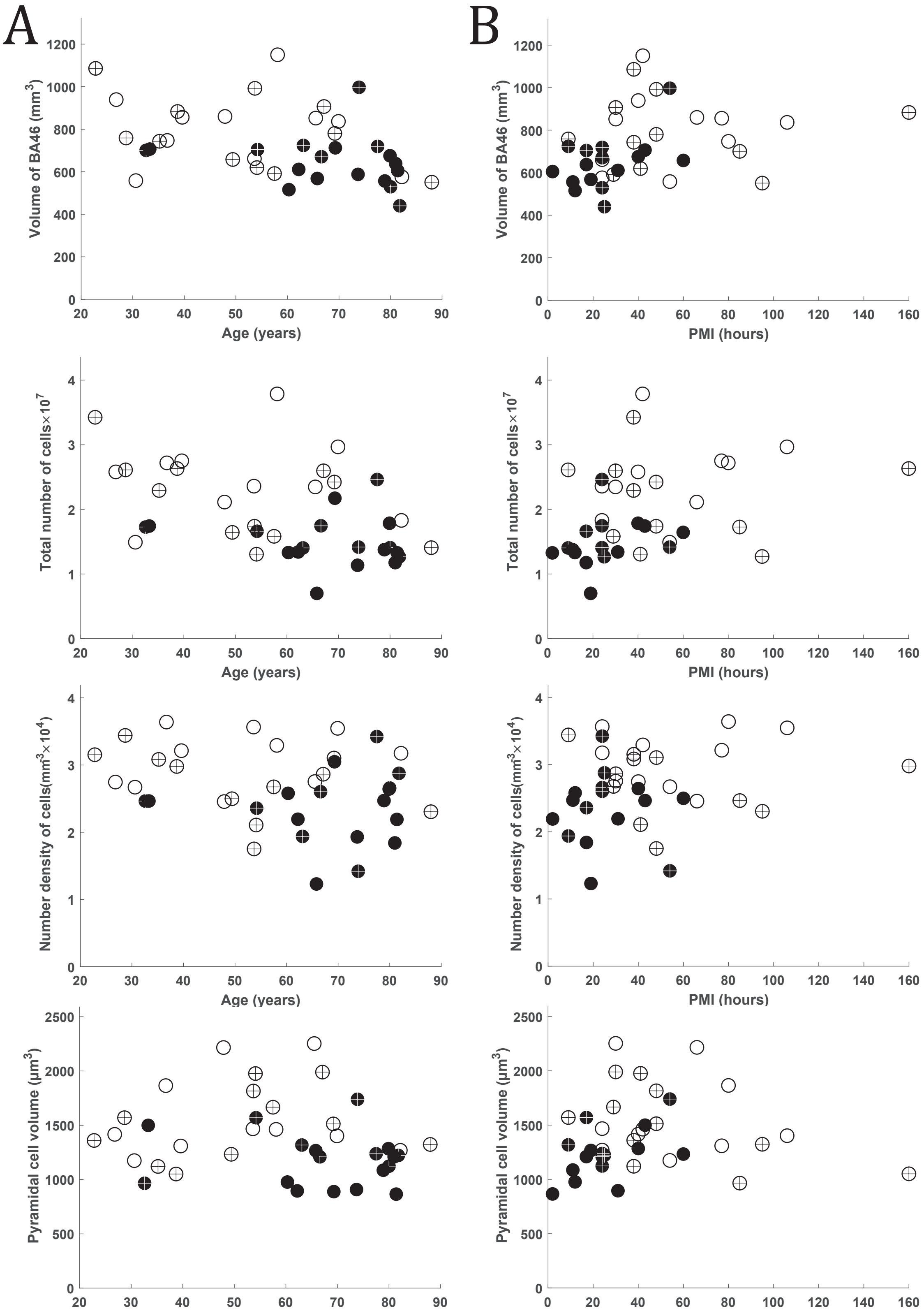

Figure S4: Scatter plot depicting the correlation of age and PMI. Scatter plots of the volume of BA46, volume of pyramidal cells in layer III, total number of neurons, and number density estimates vs. age (A) and PMI (B). C – white circles, SU – white circle with a black cross, MDD – black circle with a white cross, and SCH – black circle.

Supplementary Data Tables

Table S2: Stereological parameters for analyzing the volume area of BA46 and pyramidal cells in layer III. Before the study, a pilot test determined the sampling fraction and the 7x7 point grid size.

Study technique	Probe	Sampling faction of ROI			Lens	Point grid points/CF area	
Volume estimation	Point grid	100%	x-step	7146 μm	x1.25	Group:	1
			y-step	5337 μm		Points:	7
Optical fractionator	CF	0.3%	x-step	1040 μm	x60	CF:	3244 μm^2
			y-step	1040 μm			
Volume tensors	CF	2%	x-step	403 μm	x60	CF:	3244 μm^2
			y-step	403 μm			

Counting frame (CF), Region of interest (ROI)

Table S3: The volume of the measured pyramidal cells in layer III of BA46 based on 3D-reconstruction. Bonferroni test for pairwise comparison of the means for the four groups. Control (C), Major depressive disorder (MDD), Suicide (SU), Schizophrenia (SCH)

	Volume (m^3)			
**Group**	**Mean**	**STD**	**CV**	
C	759	147	0.19	
MDD	611	127	0.20	
SU	749	165	0.22	
SCH	573	77	0.13	
**Pairwise comparison**	**Difference of means**	**95% CI difference of means**		**p-value**
C vs MDD	149	(-29,	327)	0.15
C vs SU	10	(-154,	174)	1
C vs SCH	186	(18,	354)	0.02
MDD vs SU	-139	(-314,	36)	0.2
MDD vs SCH	37	(-141,	215)	1
SU vs SCH	176	(12,	340)	0.03

Table S5: The orientation of the measured pyramidal cells in layer III of BA46 based on 3D-reconstruction. Bonferroni test for pairwise comparison of the means for the four groups. Control (C), Major depressive disorder (MDD), Suicide (SU), Schizophrenia (SCH)

**Orientation (°)**				
**Group**	**Mean**	**STD**	**CV**	
C	29.8	3.97	0.13	
MDD	30.6	6.80	0.22	
SU	28.2	3.92	0.14	
SCH	27.8	4.02	0.14	
**Pairwise comparison**	**Difference of means**	**95% CI difference of means**		**p-value**
C vs MDD	-0.83	(-7.0,	5.4)	1
C vs SU	1.62	(-4.1,	7.3)	1
C vs SCH	2	(-3.9,	7.8)	1
MDD vs SU	2.45	(-3.6,	8.5)	1
MDD vs SCH	2.83	(-3.4,	9.0)	1
SU vs SCH	0.38	(-5.3,	6.1)	1

Table S4: The diameter of the measured pyramidal cells in layer III of BA46 based on 3D-reconstruction. Bonferroni test for pairwise comparison of the means for the four groups. Control (C), Major depressive disorder (MDD), Suicide (SU), Schizophrenia (SCH)

**Diameter (μm)**				
**Group**	**Mean**	**STD**	**CV**	
C	10.84	0.60	0.06	
MDD	10.52	0.75	0.07	
SU	10.92	0.84	0.07	
SCH	10.42	0.73	0.07	
**Pairwise comparison**	**Difference of means**	**95% CI difference of means**		**p-value**
C vs MDD	0.32	(-0.66,	1.30)	1
C vs SU	-0.08	(-0.98,	0.82)	1
C vs SCH	0.42	(-0.51,	1.35)	1
MDD vs SU	-0.40	(-1.36,	0.56)	1
MDD vs SCH	0.10	(-0.88,	1.08)	1
SU vs SCH	0.50	(-0.40,	1.40)	0.79

Table S6: The sphericity of the measured pyramidal cells in layer III of BA46 based on 3D-reconstruction. Bonferroni test for pairwise comparison of the means for the four groups. Control (C), Major depressive disorder (MDD), Suicide (SU), Schizophrenia (SCH)

**Sphericity**				
**Group**	**Mean**	**STD**	**CV**	
C	0.34	0.02	0.07	
MDD	0.35	0.03	0.08	
SU	0.33	0.02	0.07	
SCH	0.34	0.02	0.05	
**Pairwise comparison**	**Difference of means**	**95% CI difference of means**		***p*-value**
C vs MDD	-0.01	(-0.04,	0.02)	1
C vs SU	0.01	(-0.02,	0.04)	1
C vs SCH	0	(-0.03,	0.03)	1
MDD vs SU	0.02	(-0.01,	0.05)	0.43
MDD vs SCH	0.01	(-0.02,	0.04)	1
SU vs SCH	-0.01	(-0.04,	0.02)	1

Table S7: Statistical descriptors of the estimated total volume of BA46 in layer III and Bonferroni output. Control (C), Major depressive disorder (MDD), Suicide (SU), and Schizophrenia (SCH).

**Volume of BA46 in layer III** (mm^3)				
**Group**	**Mean**	**STD**	**CV**	
C	804	177	0.22	
MDD	686	163	0.23	
SU	779	172	0.22	
SCH	618	65	0.10	
**Pairwise comparison**	**Difference of means**	**95% CI difference of means**		***p*-value**
C vs MDD	118	(-83,	319)	0.65
C vs SU	25	(-160,	210)	1
C vs SCH	186	(-3,	375)	0.056
MDD vs SU	-94	(-290,	103)	1
MDD vs SCH	68	(-133,	269)	1
SU vs SCH	161	(-24,	347)	0.12

Table S8: Statistical descriptors of the estimated total number of pyramidal cells in layer III of BA46 and Bonferroni output. Control (C), Major depressive disorder (MDD), Suicide (SU), and Schizophrenia (SCH).

**Total Number**(10^6)				
**Group**	**Mean**	**STD**	**CV**	
C	24.94	6.37	0.25	
MDD	16.36	3.77	0.23	
SU	21.38	6.78	0.32	
SCH	14.08	4.07	0.29	
**Pairwise comparison**	**Difference of means**	**95% CI**	**difference of means**	***p*-value**
C vs MDD	8.59	(1.24,	15.94)	0.014
C vs SU	3.56	(-3.21,	10.33)	0.90
C vs SCH	10.86	(3.94,	17.79)	0.0006
MDD vs SU	-5.03	(-12.22,	2.17)	0.35
MDD vs SCH	2.28	(-5.07,	9.62)	1
SU vs SCH	7.30	(0.53,	14.07)	0.029

Table S9: Statistical descriptors of the estimated number density of pyramidal cells in layer III of BA46 and Bonferroni output. Control (C), Major depressive disorder (MDD), Suicide (SU), and Schizophrenia (SCH).

**Density** (mm^{-3})				
**Group**	**Mean**	**STD**	**CV**	
C	31063	4224	0.13	
MDD	24677	6009	0.24	
SU	27241	5110	0.19	
SCH	22595	5068	0.22	
**Pairwise comparison**	**Difference of means**	**95% CI**	**difference of means**	***p*-value**
C vs MDD	6385	(-362,	13133)	0.073
C vs SU	3822	(-2393,	10037)	0.57
C vs SCH	8467	(2105,	14829)	0.004
MDD vs SU	-2563	(-9173,	4046)	1
MDD vs SCH	2081	(-4666,	8829)	1
SU vs SCH	4645	(-1570,	10861)	0.26

Table S10: Statistical descriptors of the estimated average volume of pyramidal cells in layer III of BA46 and Bonferroni output. Control (C), Major depressive disorder (MDD), Suicide (SU), and Schizophrenia (SCH).

**Neuronal volume (μm^3)**			
**Group**	**Mean**	**STD CV**	
C	1232	298 0.24	
MDD	1052	166 0.16	
SU	1162	232 0.20	
SCH	880	186 0.21	
**Pairwise comparison**	**Difference of means**	**95% CI difference of means**	***p*-value**
C vs MDD	181	(-124, 485)	0.64
C vs SU	71	(-210, 351)	1
C vs SCH	352	(65, 639)	0.009
MDD vs SU	-110	(-408, 188)	1
MDD vs SCH	172	(-133, 476)	0.74
SU vs SCH	282	(1.35, 562)	0.04

Table S11: Statistical descriptors of the estimated elongation index of pyramidal cells in layer III of BA46 and Bonferroni output. Control (C), Major depressive disorder (MDD), Suicide (SU), and Schizophrenia (SCH).

**Elongation index**			
**Group**	**Mean**	**STD CV**	
C	1.31	0.12 0.09	
MDD	1.24	0.07 0.06	
SU	1.24	0.08 0.06	
SCH	1.34	0.07 0.05	
**Pairwise comparison**	**Difference of means**	**95% CI difference of means**	***p*-value**
C vs MDD	0.07	(-0.05, 0.19)	0.68
C vs SU	0.07	(-0.03, 0.18)	0.44
C vs SCH	-0.02	(-0.14, 0.08)	1
MDD vs SU	0.003	(-0.11, 0.12)	1
MDD vs SCH	-0.09	(-0.21, 0.02)	0.22
SU vs SCH	-0.10	(-0.21, 0.01)	0.12

Table S12: Statistical descriptors of estimated nucleus displacement of pyramidal cells in layer III of BA46 and Bonferroni output. Control (C), Major depressive disorder (MDD), Suicide (SU), and Schizophrenia (SCH).

**Displacement (μm)**				
**Group**	**Mean**	**STD**	**CV**	
C	0.66	0.29	0.43	
MDD	0.53	0.11	0.21	
SU	0.78	0.40	0.51	
SCH	0.40	0.25	0.61	
**Pairwise comparison**	**Difference of means**	**95% CI difference of means**		**p-value**
C vs MDD	0.13	(-0.26,	0.52)	1
C vs SU	-0.12	(-0.48,	0.24)	1
C vs SCH	0.26	(-0.11,	0.62)	0.32
MDD vs SU	-0.25	(-0.63,	0.13)	0.43
MDD vs SCH	0.13	(-0.26,	0.51)	1
SU vs SCH	0.38	(0.02,	0.73)	0.03

Supplementary Note 3

Point pattern analysis

The data consists of 39 point patterns containing 3D coordinates for the locations of centroids of pyramidal cells in layer III of BA46. Such a point pattern is considered as a realisation of a stochastic mechanism called a point process. Each point pattern corresponds to a subject, and each subject belongs to one of the four groups, control, depression, schizophrenia, and suicide. There are 10 subjects in the control group (C), 8 subjects who suffered from major depressive disorder (MDD), 10 subjects who suffered from schizophrenia (SCH), and 11 subjects who had committed suicide with a history of depression (SU).

The cylindrical K-function

To detect columnar structures in the point patterns, we used the cylindrical K-function, which was introduced in [2], and has previously been used to analyze the spatial structure of pyramidal cells (see e.g.[3], [4],[5]). The cylindrical K-function depends on a direction u, a radius r, and a height t. We use the notation $K_u(r,t)$ for the cylindrical K-function and ρ for the intensity of the underlying point process (the expected number of points per unit volume, see Fig.S5).

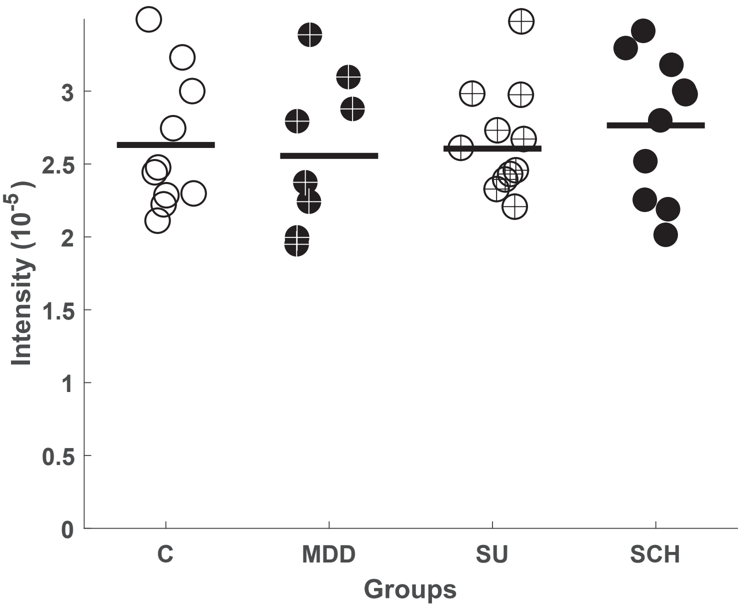

Figure S5: Estimated intensity for each point pattern plotted by group. Control (C), Major depressive disorder (MDD), Suicide (SU), and Schizophrenia (SCH).

To understand the intuitive interpretation of $K_u(r,t)$, consider a cylinder C_v in 3D space with direction u, base radius r, and height $2t$ which is centred at a randomly selected point v of the underlying point process X. Then, $\rho K_u(r,t)$ is the expected number of further points from X within C_v. If a point process exhibits a columnar structure in a specific direction, $K_u(r,t)$ is expected to be particularly high for some range of r and t values when u is that direction. For estimating $K_u(r,t)$ from a point pattern, we use the nonparametric estimate from [2]. For the choice of u, we considered the directions of the three main axes; for the choice of r, we considered 128 equidistant points in the interval from $0\mu m$ to $25\mu m$; and for t we used $t = 80$. Note that when trying to detect a columnar structure, it makes sense to use $r < t$.

An important assumption for using the cylindrical K-function is that the underlying point process is homogeneous. We assessed this assumption from the observed point patterns by looking at the histograms of each coordinate and 2D projections. All point patterns appeared reasonably homogeneous.

To use the estimates $\hat{K}_u(r,t)$ of the cylindrical K-function to make statements about columnar structures in the data, we compared the obtained estimates with the situation of a so-called homogeneous Poisson process also referred to as 'complete spatial randomness' (CSR). As the name suggests,

CSR is the situation where there is no structure in data. We make the comparison based on a number of simulated point patterns under CSR. The estimate of the cylindrical K-function is calculated for each of these simulations and the resulting curves are ordered by means of the so-called extreme rank length measure [6, 7]. The ordered curves can then be used to construct a global envelope test for the null hypothesis that the observed point pattern is a realisation of CSR as described in [6]. We used a 95% global envelope test and 2000 simulations under CSR (the number of simulations followed the recommendations in the above references). The method returns a p-value and a graphical interpretation in the form of a global envelope which consists of a region in which the observed curve falls completely if and only if the global envelope test cannot be rejected at approximately level 5%. If the empirical curve falls above the envelope for some range of r-values, it indicates that the estimate is higher than expected under CSR, which in turn indicates some clustering. If this behaviour is most pronounced for a specific direction u, it indicates cylinder-shaped clusters in this direction. If the curve falls below the envelope, it is smaller than expected under CSR, which indicates the repulsion between points.

Figures S6, S7, S8, and S9 show the cylindrical K-function for all three directions for each subject plus 95% global envelopes under CSR and the p-values of the corresponding tests. For the majority of subjects, and in particular for all 10 control subjects, 7 in MDD, 7 in SCH, and 3 in SU, there is clear evidence of a columnar structure in the direction of the x-axis, especially when considering radii between 5 and $20\mu m$. In some of the point patterns, there is some evidence of repulsive behavior between points, which is very intuitive since each cell occupies an amount of physical space, which is not accounted for, and the cells cannot overlap. However, this may not explain all repulsion.

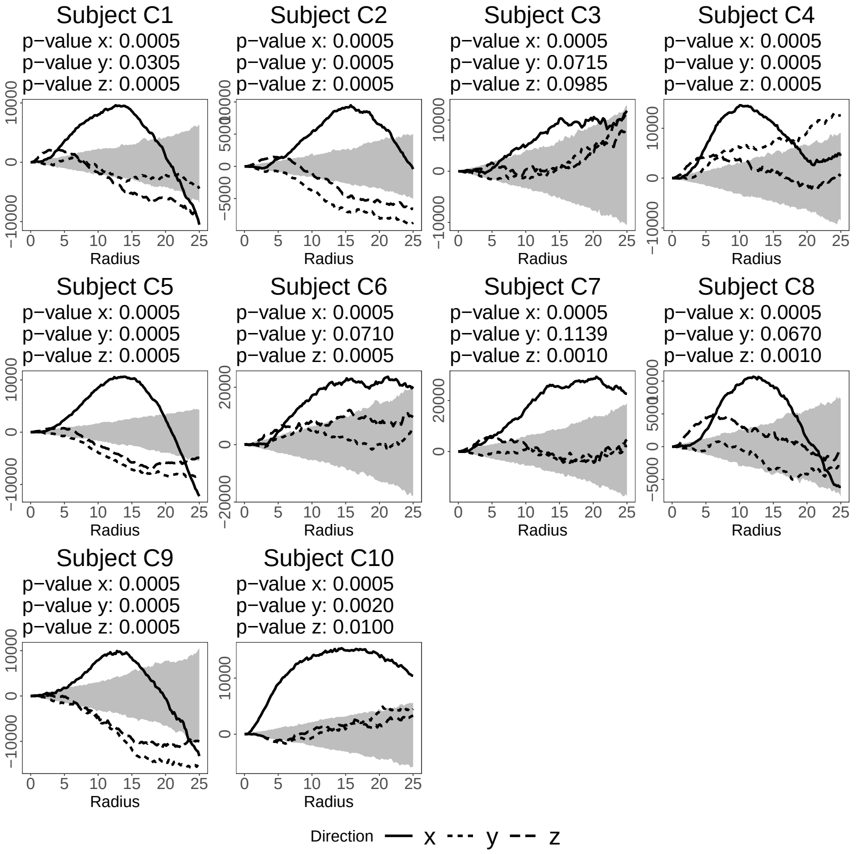

Figure S6: The curves show the cylindrical K-function minus its theoretical value under CSR in each of the directions indicated by the legend and a 95% global envelope under CSR plus the p-values of the corresponding global envelope tests. Each plot corresponds to a subject in the control group.

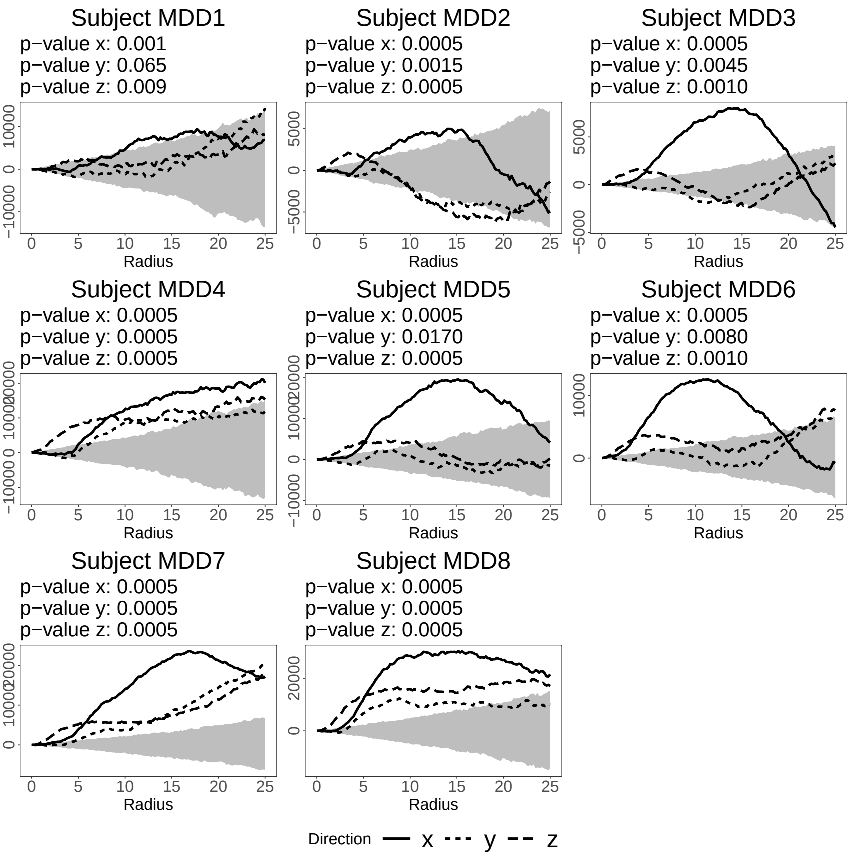

Figure S7: The curves show the cylindrical K-function minus its theoretical value under CSR in each of the directions indicated by the legend and a 95% global envelope under CSR plus the p-values of the corresponding global envelope tests. Each plot corresponds to a subject in the major depressive disorder group.

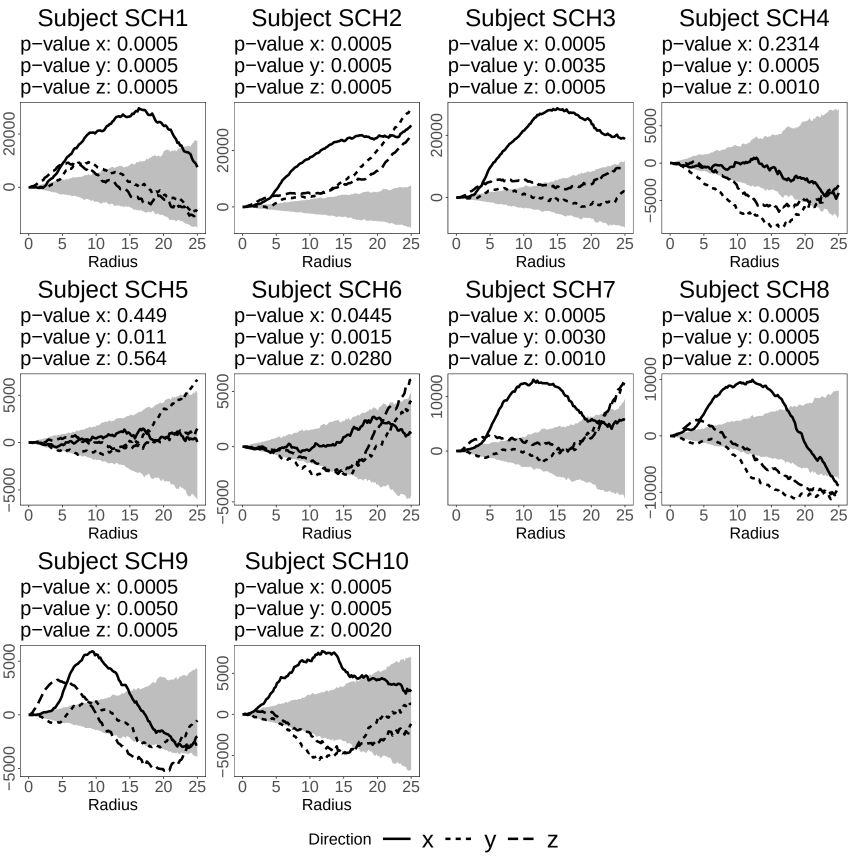

Figure S8: The curves show the cylindrical K-function minus its theoretical value under CSR in each of the directions indicated by the legend and a 95% global envelope under CSR plus the p-values of the corresponding global envelope tests. Each plot corresponds to a subject in the schizophrenia group.

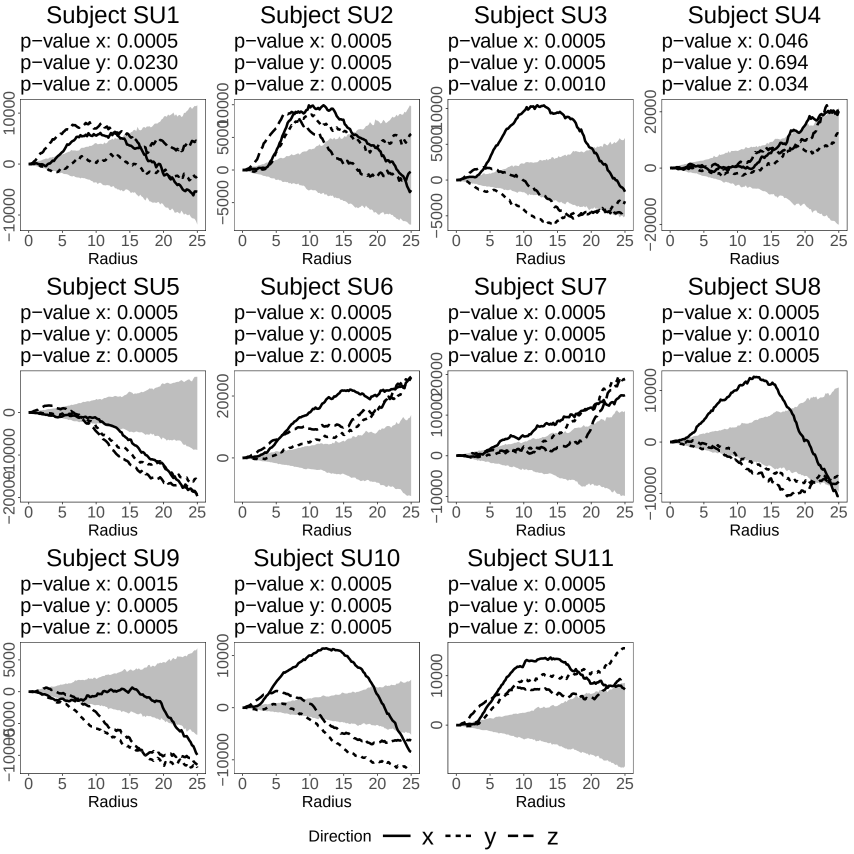

Figure S9: The curves show the cylindrical K-function minus its theoretical value under CSR in each of the directions indicated by the legend and a 95% global envelope under CSR plus the p-values of the corresponding global envelope tests. Each plot corresponds to a subject in the suicide group.

To get a better visualisation for comparison, Figure S10 shows all empirical cylindrical K-functions in the same plot split by direction and group. Notice that there is a lot of variation in the empirical cylindrical K-function between subjects, and it is difficult to spot whether there is a clear difference between groups.

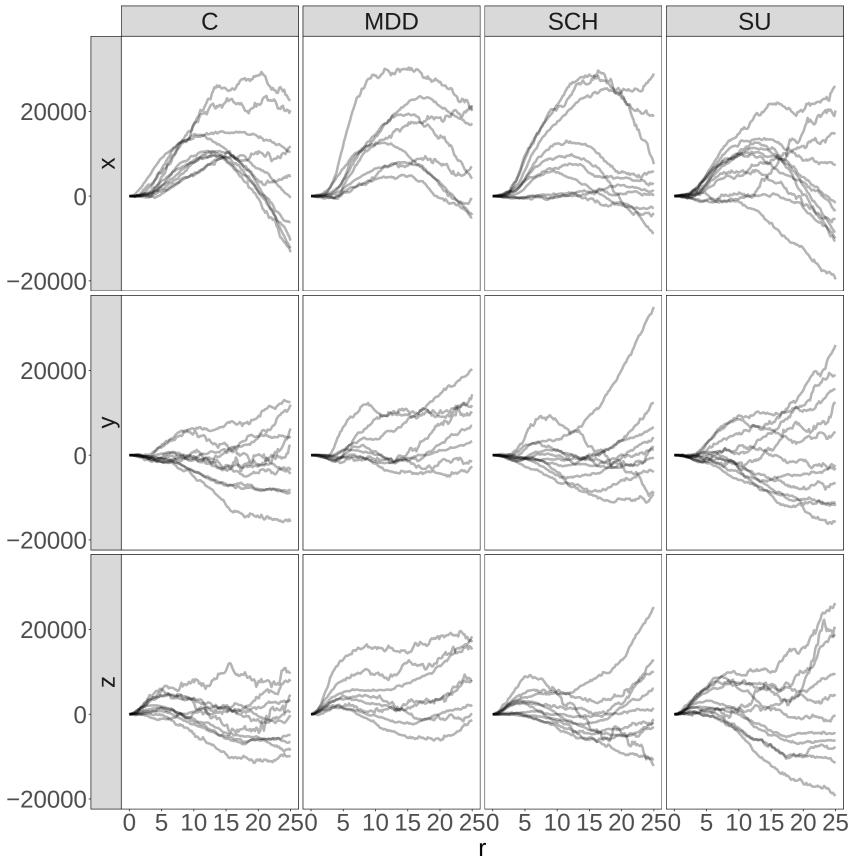

Figure S10: The curves show the cylindrical K-function minus its theoretical value under CSR in each of the directions stated at the left. Each curve corresponds to a subject in the group stated at the top. Control (C), Major depressive disorder (MDD), Suicide (SU), and Schizophrenia (SCH).

To summarise the general behaviour of the empirical cylindrical K-function within each group, we pool the estimates in the following way. Let $\hat{K}_u^{ij}(r,t)$ be the empirical cylindrical K-function of the $i'th$ point pattern in the $j'th$ group and let this point pattern have n_{ij} points. Let there be m_j point patterns in group j. To estimate the cylindrical K-function for group j, we use the weighted mean

$$\hat{K}_u^j(r,t) = \frac{\sum_{i=1}^{m_j} n_{ij}\hat{K}_u^{ij}(r,t)}{\sum_{i=1}^{m_j} n_{ij}}. \tag{1}$$

This weighted mean was recommended for estimating Ripley's K-function (see below) from replicated point patterns in [8] (both when the windows are the same and different sizes and when there is a common and pattern-specific

intensity), and the argumentation seems reasonable for the cylindrical K-function as well. Figure S11 shows these estimates for each group in the three considered directions. This confirms that there is an overall tendency for the cylindrical K-function in the direction of the x-axis to reach particularly high values, which indicates a columnar structure in this direction.

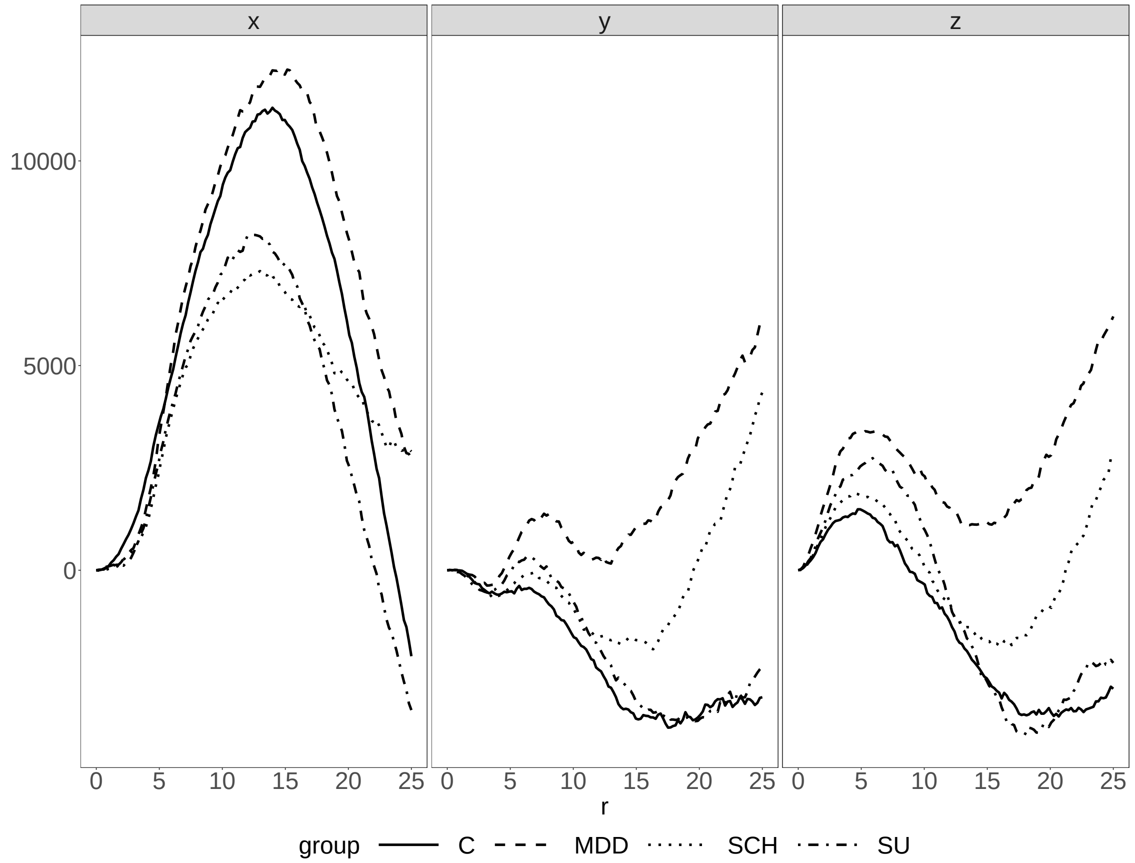

Figure S11: Weighted mean of the empirical cylindrical K-functions minus the theoretical value under CSR in the direction indicated at the top of each plot for each group as specified in the legend. Control (C), Major depressive disorder (MDD), Suicide (SU), and Schizophrenia (SCH).

To decide whether the apparent differences between the empirical cylindrical K-functions estimated for each group are significant, we use random permutations to construct a global envelope test to get an approximate test of the null hypothesis that all point patterns are realisations of spatial point processes with the same cylindrical K-function in the following way. Denote the number of groups by g. By concatenating $\hat{K}_u^1, \dots, \hat{K}_u^g$, one curve of data is achieved. This curve is then compared to the situation under the null hy-

pothesis by making a number of random permutations of the point patterns; recalculating $\hat{K}_u^j(r,t)$ for each group, which no longer consists of the same point patterns but has the same number of point patterns; concatenating the estimates; and finally using these curves to construct a global envelope test as above. We considered a 95% global envelope test based on 8000 random permutations. The observed concatenated curve will fall completely within the envelope if and only if the null hypothesis of no difference in the cylindrical K-function between groups cannot be rejected at level 5%. If the concatenated curves fall outside the envelope for any r-value, the test is rejected, and there are significant differences between the groups. The results of such tests for the cylindrical K-function directed along one of the three main axes can be seen in Figures S12, S13, and S14 where we for better visualisation split the concatenated curves into one plot for each group. These tests demonstrate that the cylindrical K-function along the x, y, and z-axes did not detect any significant difference between the groups.

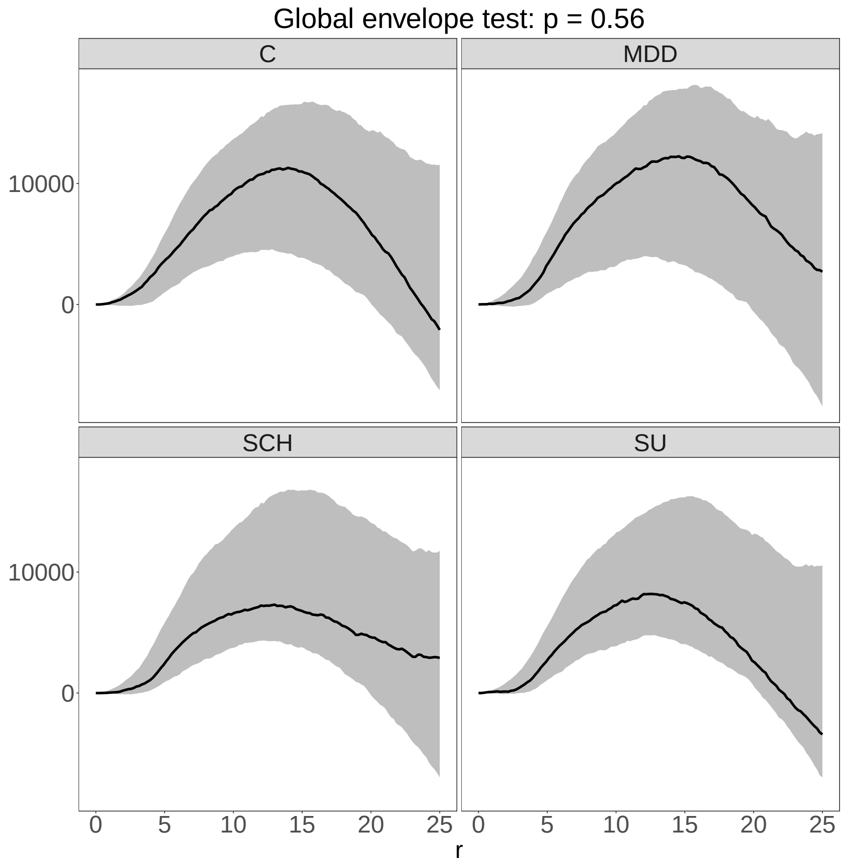

Figure S12: 95% global envelopes (gray area) based on 8000 random permutations for testing whether there are significant differences between the cylindrical K-functions in the direction of the x-axis of the groups. All curves have been subtracted from the theoretical value under CSR. The solid curves correspond to the weighted means of data. The group is indicated at the top of each plot, and the p-value of the test is stated at the top of the figure. Control (C), Major depressive disorder (MDD), Suicide (SU), and Schizophrenia (SCH).

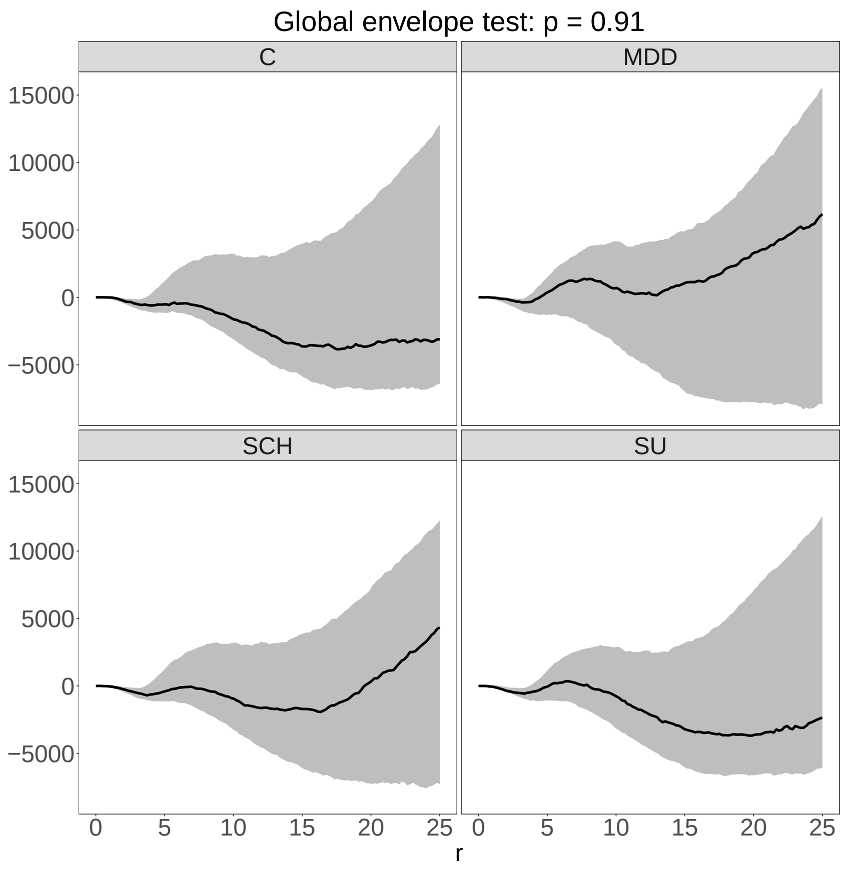

Figure S13: 95% global envelopes (gray area) based on 8000 random permutations for testing whether there are significant differences between the cylindrical K-functions in the direction of the y-axis of the groups. All curves have been subtracted from the theoretical value under CSR. The solid curves correspond to the data. The group is indicated at the top of each plot, and the p-value of the test is stated at the top of the figure. Control (C), Major depressive disorder (MDD), Suicide (SU), and Schizophrenia (SCH).

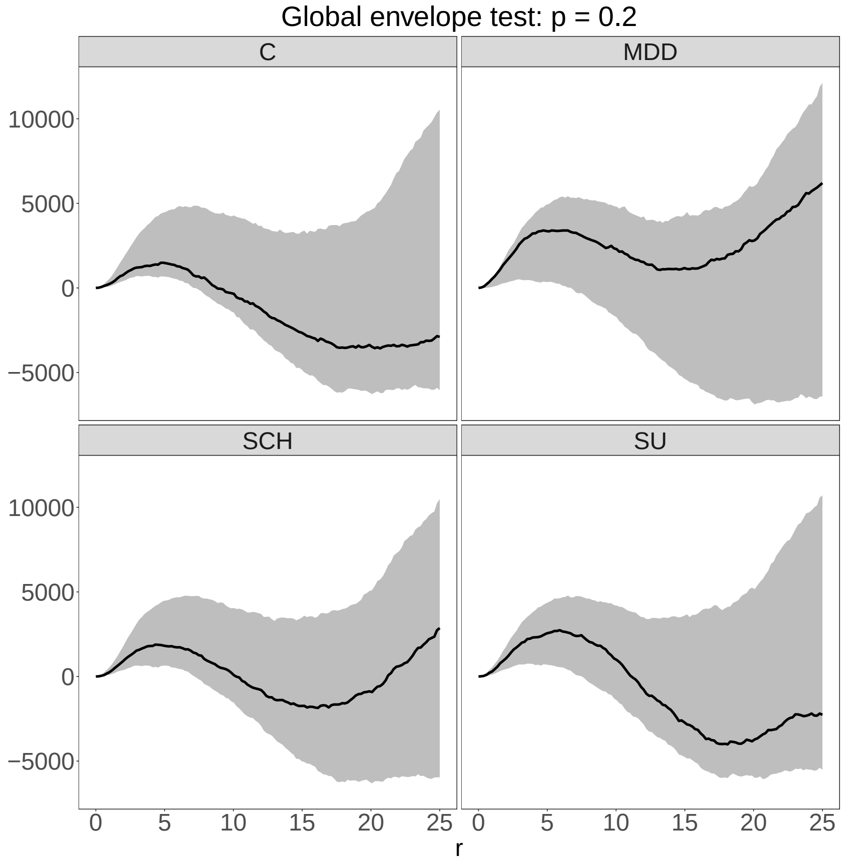

Figure S14: 95% global envelopes (gray area) based on 8000 random permutations for testing whether there are significant differences between the cylindrical K-functions in the direction of the z-axis of the groups. All curves have been subtracted from the theoretical value under CSR. The solid curves correspond to the data. The group is indicated at the top of each plot, and the p-value of the test is stated at the top of the figure. The red dots indicate where the curve falls outside the global envelope. Control (C), Major depressive disorder (MDD), Suicide (SU), and Schizophrenia (SCH).

Ripley's K-function

Ripley's K-function is a popular functional summary statistic for spatial point processes. It depends on an argument r, and we denote it by $K(r)$. If ρ is the intensity of the process, $\rho K(r)$ is interpreted as the expected number of further points within the distance r of a typical point of the point process. Note that this interpretation is similar to the interpretation of the cylindrical K-function, where we just consider a cylinder instead of a sphere. Ripley's K-function may detect some different structures in data than the cylindrical K-function. If $K(r)$ is higher (lower) than it is under CSR, it suggests that the point process is more clustered (repulsive) at interpoint distances r. When

using Ripley's K-function, we again assume homogeneity. For estimating $K(r)$ from the data, we used the translation correction estimator [9, 10] and we compared these estimates to the case of CSR with global envelopes as described for the cylindrical K-function . Figures S15, S16, S17, and S18 show the estimates minus the theoretical value under CSR and 95% global envelope for each subject. In all point patterns we see some deviations from CSR. Most point patterns show repulsive behaviour for a range of r-values, which is again very intuitive because the cells do not overlap. However, since we also see a repulsion for radii much higher than the approximate diameter of a cell, which is about $10\mu m$, it suggests that not all repulsion can be explained by the fact that the cells do not overlap. We also see evidence of clustering in about three-quarters of the subjects, especially for radii in the range of $5 - 10\mu m$.

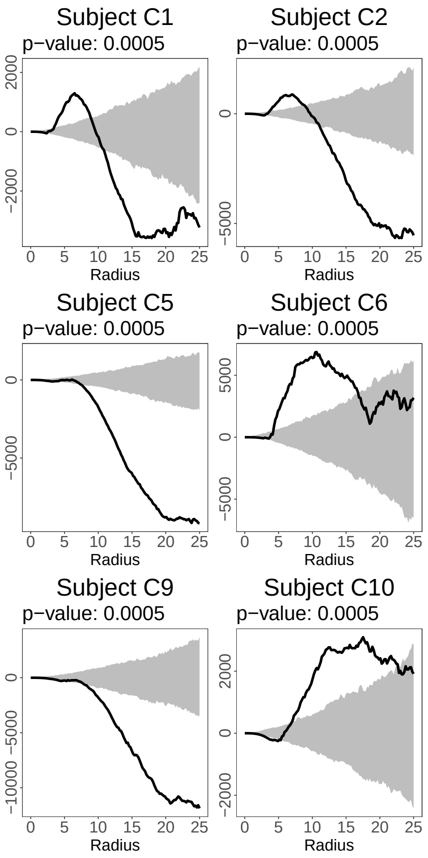

Figure S15: The plots show Ripley's K-function minus its theoretical value under CSR and a 95% global envelope (grey area) plus the p-values of the corresponding global envelope tests. Each plot corresponds to a subject in the control group.

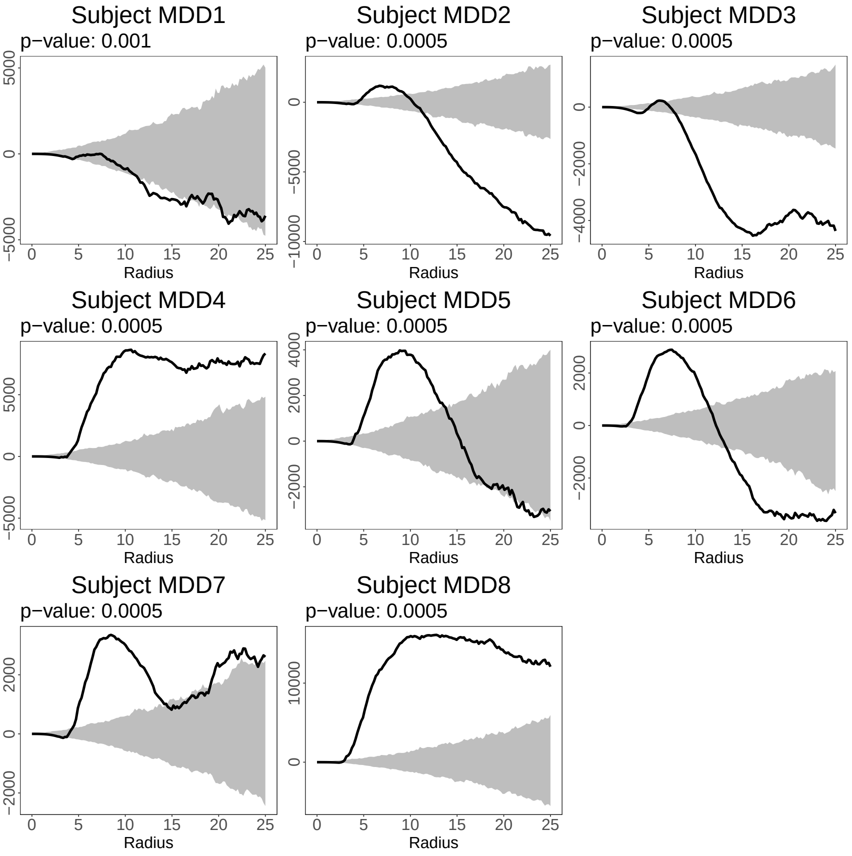

Figure S16: The plots show Ripley's K-function minus its theoretical value under CSR and a 95% global envelope (grey area) plus the p-values of the corresponding global envelope tests. Each plot corresponds to a subject in the major depressive disorder group.

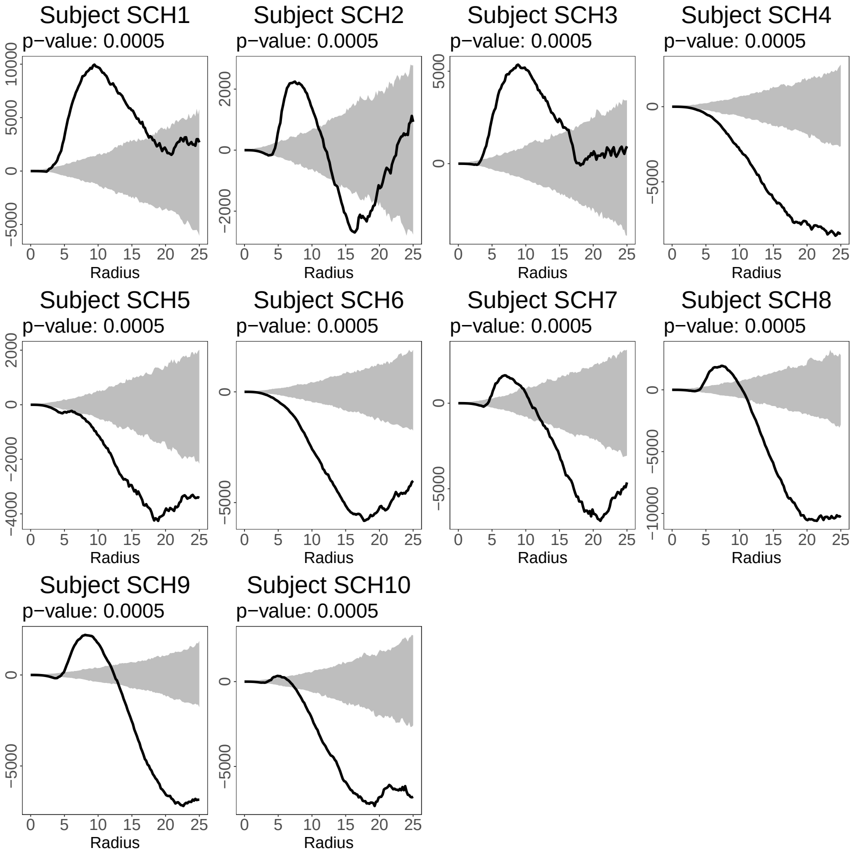

Figure S17: The plots show Ripley's K-function minus its theoretical value under CSR and a 95% global envelope (grey area) plus the p-values of the corresponding global envelope tests. Each plot corresponds to a subject in the schizophrenia group.

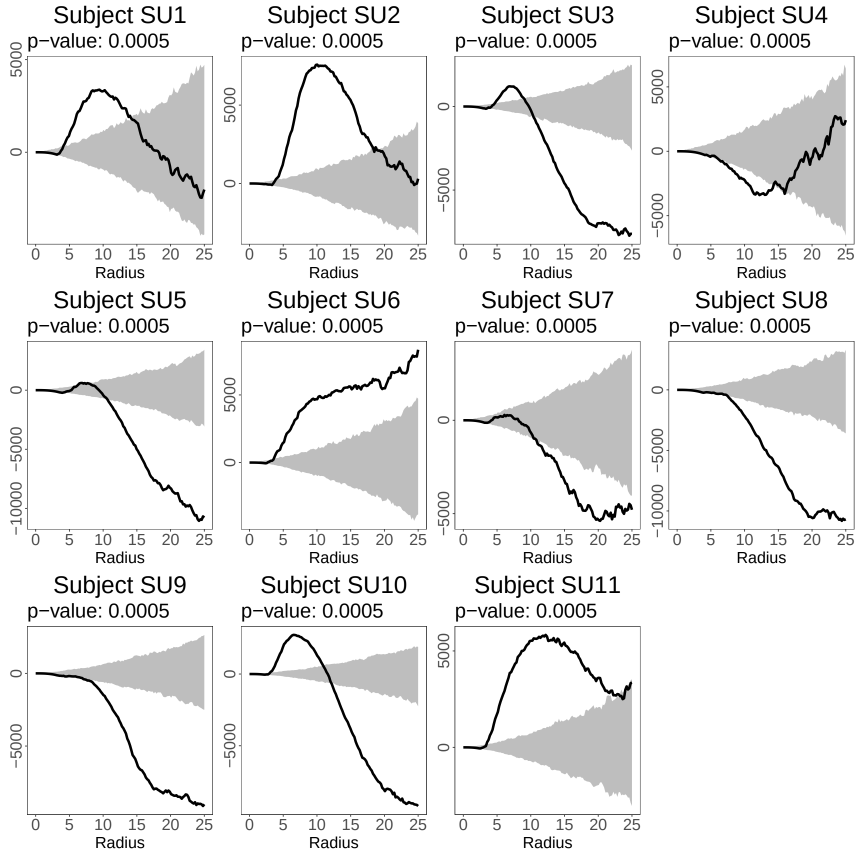

Figure S18: The plots show Ripley's K-function minus its theoretical value under CSR and a 95% global envelope (grey area) plus the p-values of the corresponding global envelope tests. Each plot corresponds to a subject in the suicide group.

Figure S19 shows all estimates of Ripley's K-function split by group. We again see a lot of variation between subjects and no clear differences between groups.

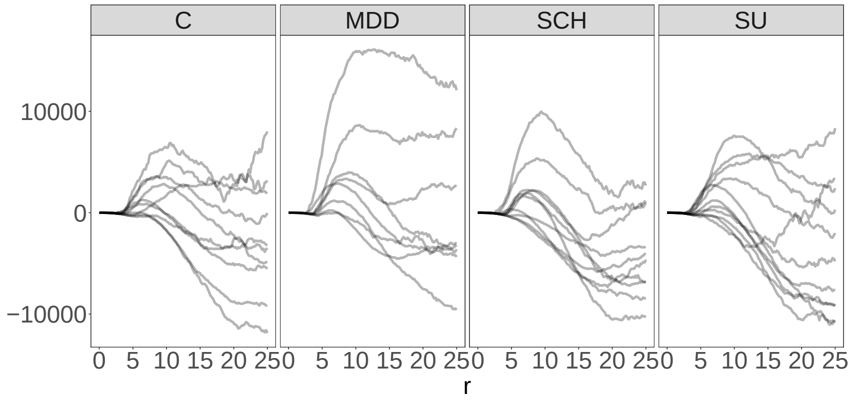

Figure S19: The curves show Ripley's K-function minus its theoretical value under CSR. Each curve corresponds to a subject in the group stated at the top. Control (C), Major depressive disorder (MDD), Suicide (SU), and Schizophrenia (SCH).

To summarise the empirical curves from each group, we used (1) with the estimate of the cylindrical K-function replaced by the estimate of Ripley's K-function, and we thus follow the recommendation in [8]. Figure S20 shows the resulting curves, which look very similar across groups.

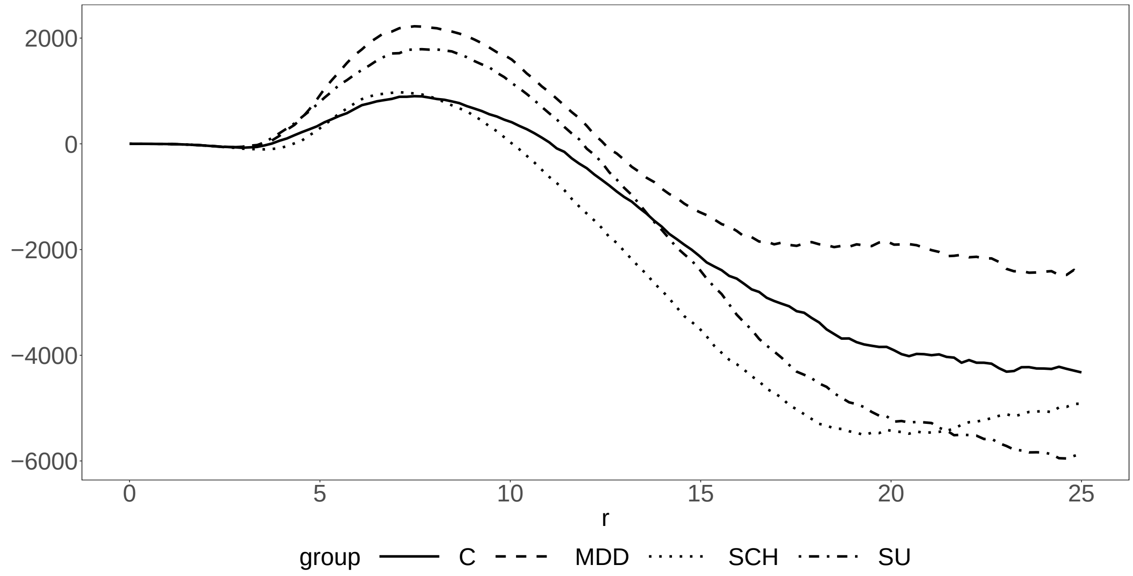

Figure S20: Weighted mean of the estimates of Ripley's K-function minus the theoretical value under CSR for each group as specified in the legend. Control (C), Major depressive disorder (MDD), Suicide (SU), and Schizophrenia (SCH).

To test whether there are significant differences in Ripley's K-function between the groups, we used a 95% global envelope test in the same way as ex-

plained for the cylindrical K-function. The result can be seen in Figure S21, which shows no significant differences in Ripley's K-function between groups.

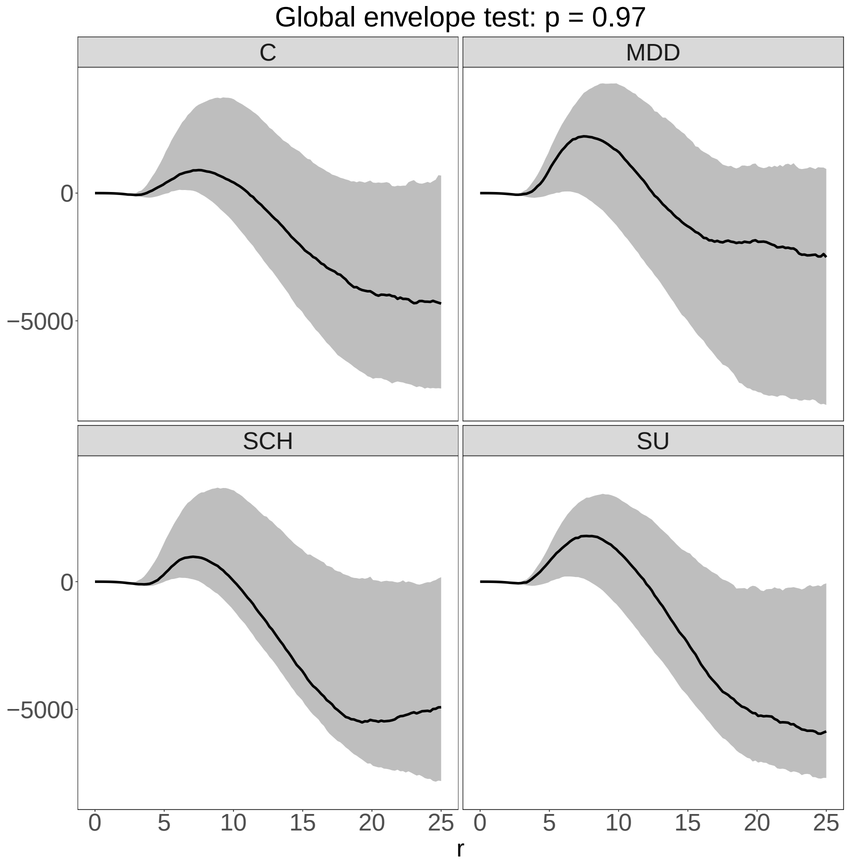

Figure S21: 95% global envelopes (gray area) based on 8000 random permutations for testing whether there are significant differences between Ripley's K-function. All curves have been subtracted from the theoretical value under CSR. The solid curves correspond to the data. The group is indicated at the top of each plot, and the p-value of the test is stated at the top of the figure. Control (C), Major depressive disorder (MDD), Suicide (SU), and Schizophrenia (SCH).

The empty-space function F

The empty-space function is a functional summary statistic of spatial point processes which is based on interpoint distances. It depends on an argument r, and we use the notation $F(r)$. The function $F(r)$ is interpreted as the probability of finding a point from the process within a sphere of radius r centered at a fixed location in space. When using the F-function, we again assume homogeneity. For estimating $F(r)$ from the data, we used the reduced sample estimator [9] and we compared these estimates to the case

of CSR with global envelopes as described for the cylindrical K-function. Figures S22, S23, S24, and S25 show the estimates and 95% global envelopes for each subject. The envelopes are in all cases very narrow. The F-function detects deviations from CSR in 28 of the point patterns.

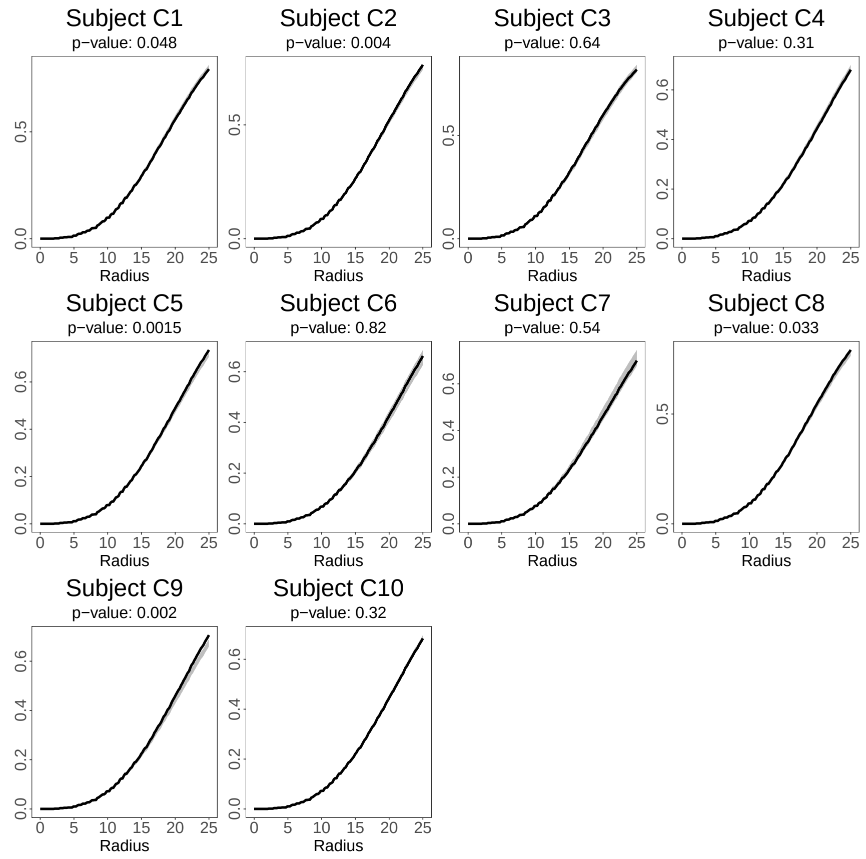

Figure S22: The plots show the F-function and a 95% global envelope (gray area) plus the p-values of the corresponding global envelope tests (at the top of each plot). Each plot corresponds to a subject in the control group.

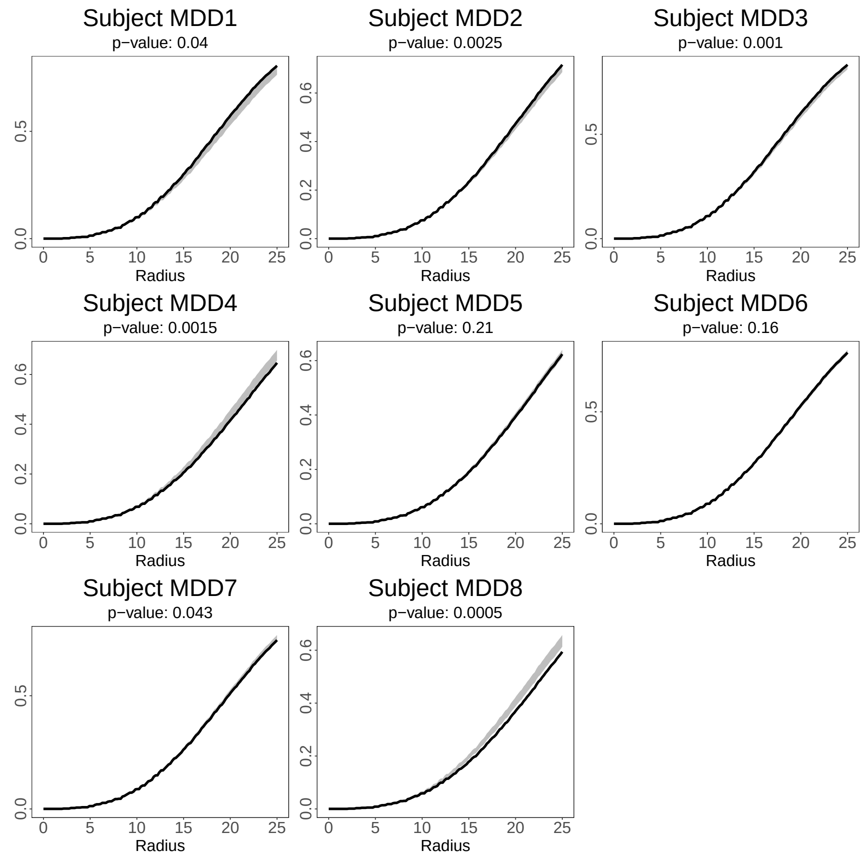

Figure S23: The plots show the F-function and a 95% global envelope (gray area) plus the p-values of the corresponding global envelope tests (at the top of each plot). Each plot corresponds to a subject in the major depressive disorder group.

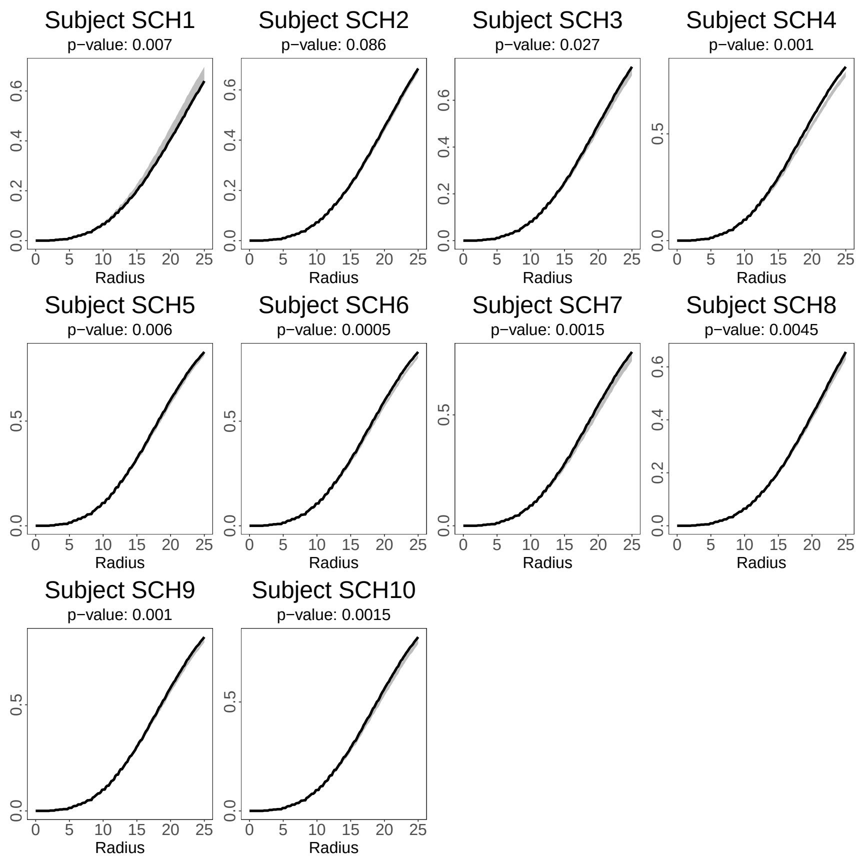

Figure S24: The plots show the F-function and a 95% global envelope (gray area) plus the p-values of the corresponding global envelope tests (at the top of each plot). Each plot corresponds to a subject in the schizophrenia group.

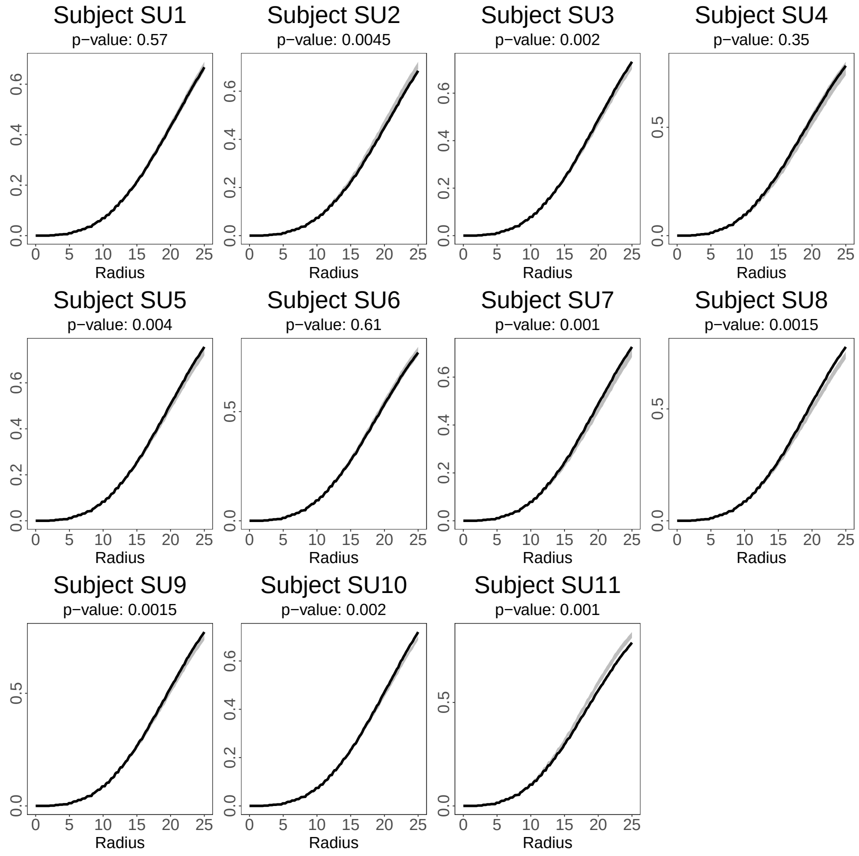

Figure S25: The plots show the F-function and a 95% global envelope (gray area) plus the p-values of the corresponding global envelope tests (at the top of each plot). Each plot corresponds to a subject in the suicide group.

Figure S26 shows all estimates of the F-function split by group. It is again difficult to spot any clear differences between groups.

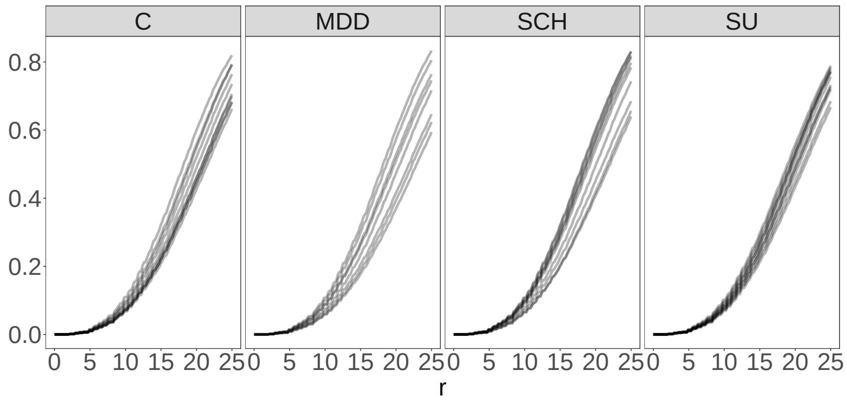

Figure S26: Each curve corresponds to the F-function of a subject in the group stated at the top. Control (C), Major depressive disorder (MDD), Suicide (SU), and Schizophrenia (SCH).

To get an estimate $\hat{F}_j(r)$ of the F-function for the j'th group, we make the weighted average in equation (19) of [9]. It only makes sense to consider one estimate of F for each group if all point patterns within a group are assumed to be realisations of point processes with the same intensity. Based on Figure S5, this appears to be a reasonable assumption. Note that this assumption was not necessary in the corresponding analyses using the cylindrical K-function and Ripley's K-function. Figure S27 shows the resulting curves, which do not show much difference between groups.

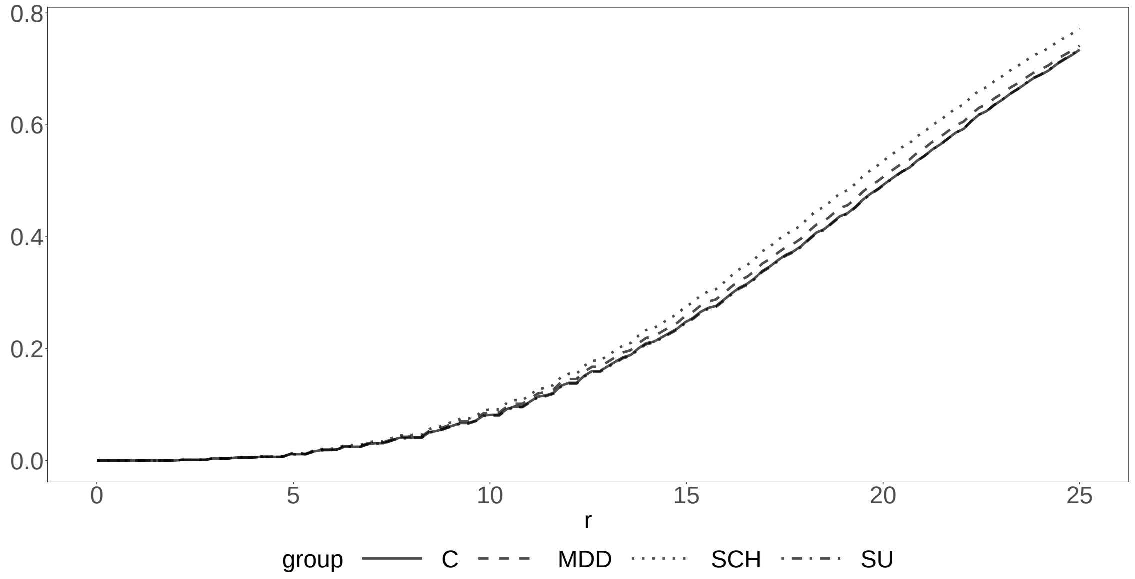

Figure S27: Weighted mean of the estimates of the F-function for each group as specified in the legend. Control (C), Major depressive disorder (MDD), Suicide (SU), and Schizophrenia (SCH).

To test whether there are significant differences in the F-function between the groups, we again used a 95% global envelope test in the same way as explained for the cylindrical K-function. The result can be seen in Figure S28, which shows no significant differences.

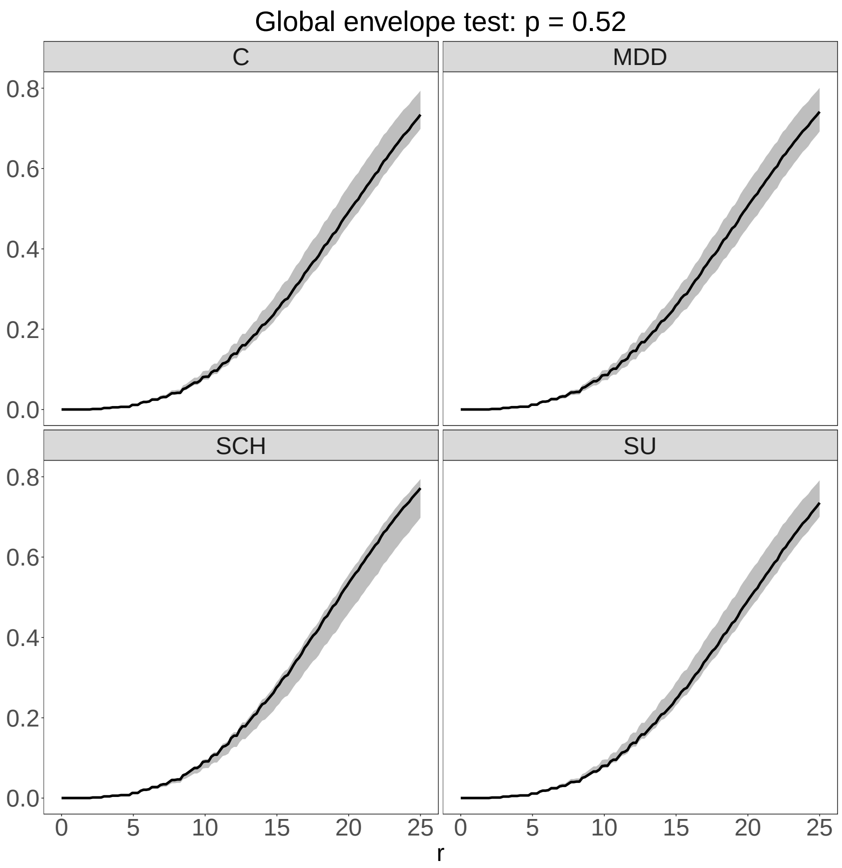

Figure S28: 95% global envelopes (gray area) based on 8000 random permutations for testing whether there are significant differences between the F-functions. The solid curves correspond to the data. The group is indicated at the top of each plot, and the p-value of the test is stated at the top of the figure. Control (C), Major depressive disorder (MDD), Suicide (SU), and Schizophrenia (SCH).

The nearest-neighbour function G

The nearest-neighbour function is again a functional summary statistic of spatial point processes which is based on interpoint distances. It depends on an argument r, and we use the notation $G(r)$. Intuitively, $G(r)$ can be interpreted as the probability of finding another point of the process within a sphere of radius r centered at a random point of the process. Thus, if $G(r)$ is higher (lower) than it is under CSR, it suggests that the point process is more clustered (repulsive) at interpoint distances r. When using the G-function, we again assume homogeneity. For estimating $G(r)$ from the data, we used the reduced sample estimator [9] and we compared these estimates to the case of CSR with global envelopes as described for the cylindrical K-

function. Figures S29, S30, S31, and S32 show the estimates and 95% global envelopes for each subject. The G-function detects deviations from CSR in all point patterns. The overall conclusions about clustering and repulsion are very similar to those obtained with Ripley's K-function.

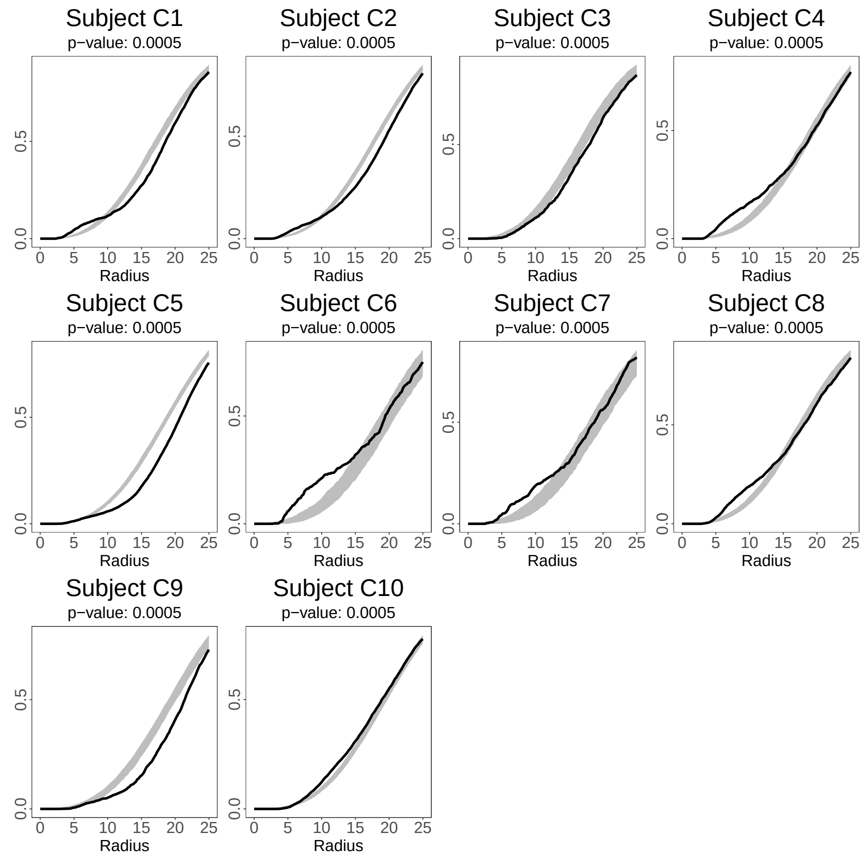

Figure S29: The plots show the G-function and a 95% global envelope (gray area) plus the p-values of the corresponding global envelope tests (at the top of each plot). Each plot corresponds to a subject in the control group.

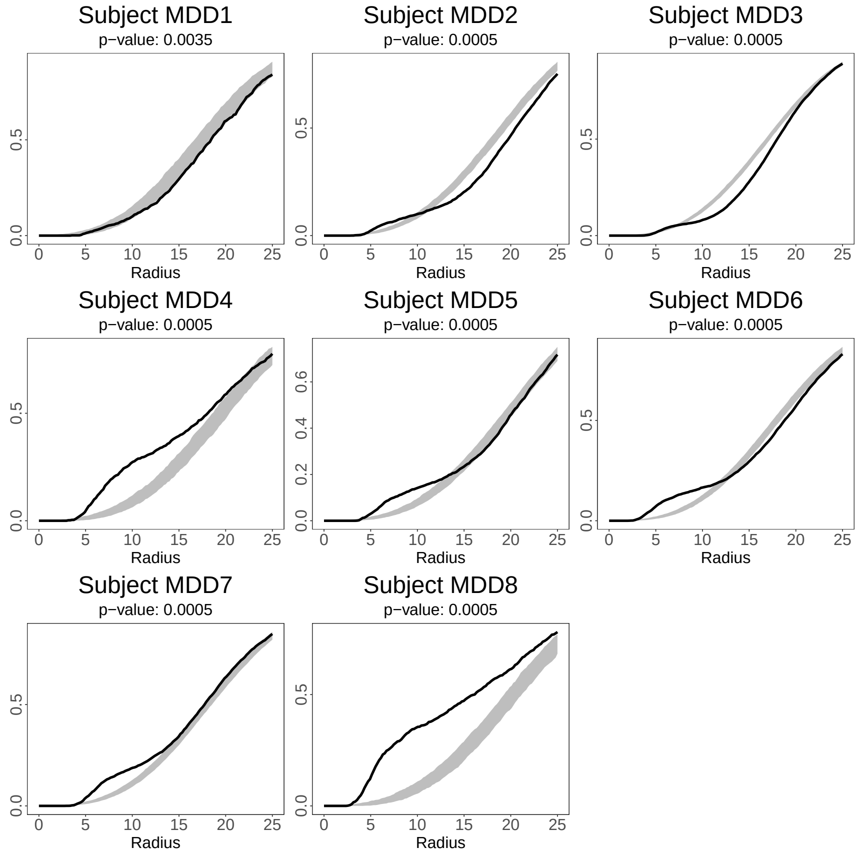

Figure S30: The plots show the G-function and a 95% global envelope (gray area) plus the p-values of the corresponding global envelope tests (at the top of each plot). Each plot corresponds to a subject in the major depressive disorder group.

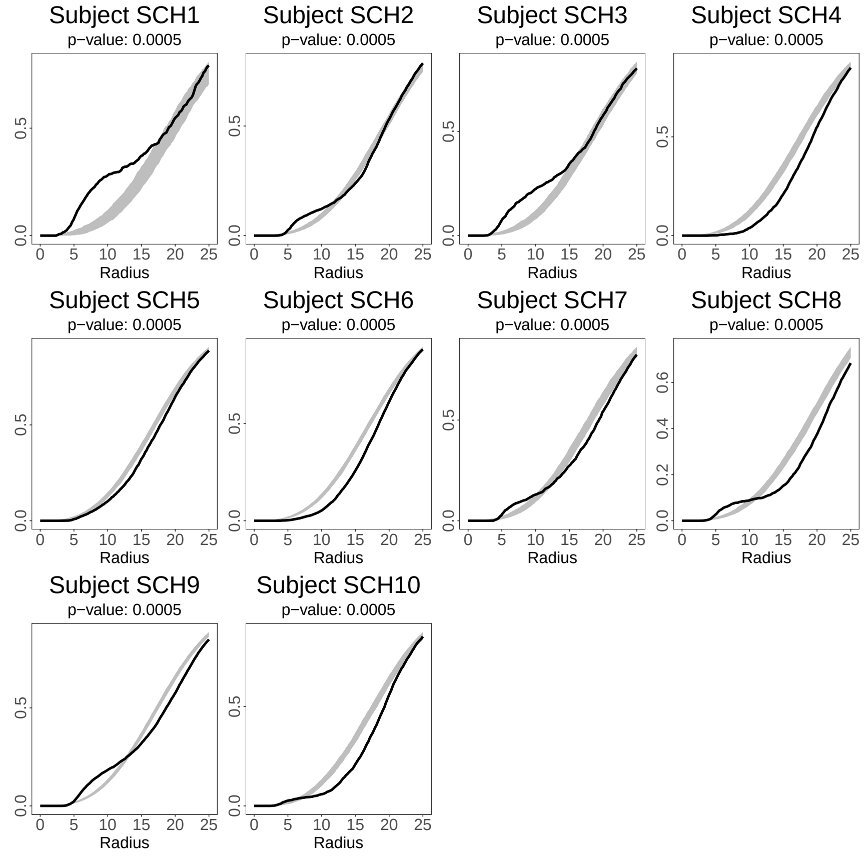

Figure S31: The plots show the G-function and a 95% global envelope (gray area) plus the p-values of the corresponding global envelope tests (at the top of each plot). Each plot corresponds to a subject in the schizophrenia group.

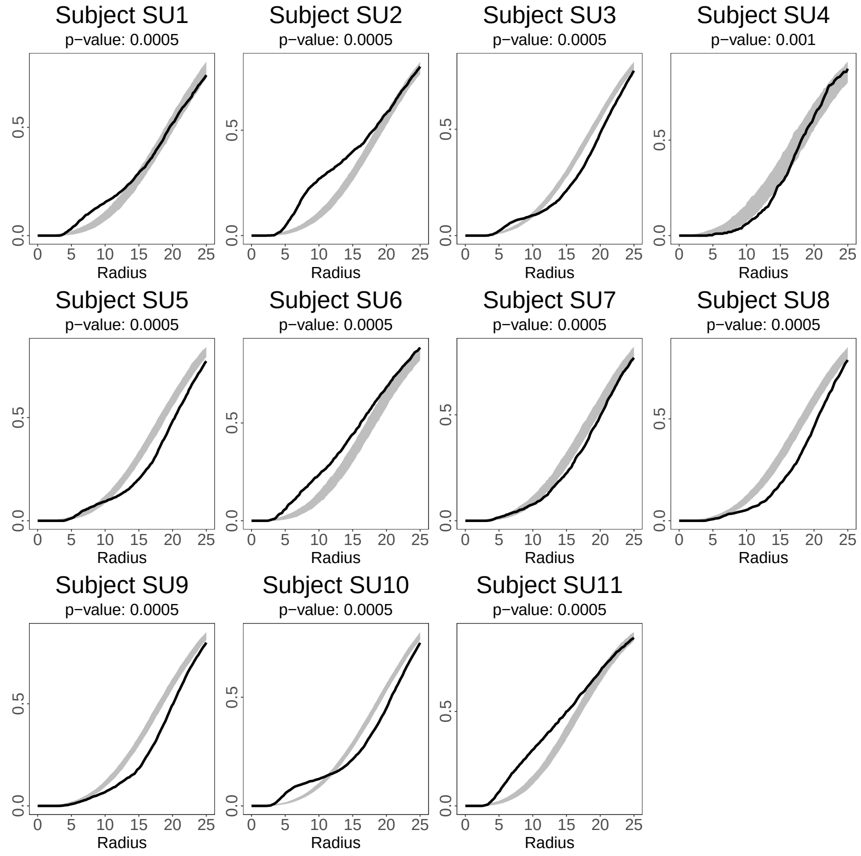

Figure S32: The plots show the G-function and a 95% global envelope (gray area) plus the p-values of the corresponding global envelope tests (at the top of each plot). Each plot corresponds to a subject in the suicide group.

Figure S33 shows all estimates of the G-function split by group. It is again difficult to spot any clear differences between groups.

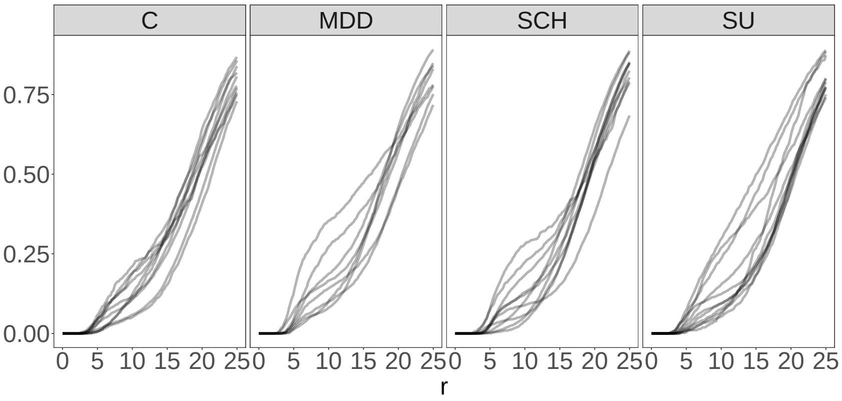

Figure S33: Each curve corresponds to the G-function of a subject in the group stated at the top. Control (C), Major depressive disorder (MDD), Suicide (SU), and Schizophrenia (SCH).

To get an estimate $\hat{G}_j(r)$ of the G-function for the j'th group, we make the weighted average

$$\hat{G}_j(r) = \frac{\sum_{i=1}^{m_j} n_{ij}^2 \hat{G}_{ij}(r)}{\sum_{i=1}^{m_j} n_{ij}^2}$$

as recommended in [11]. Here $\hat{G}_{ij}(r)$ is the estimate of the G-function obtained from the i'th point pattern in the j'th group, and the remaining notation is as in (1). It again only makes sense to consider one estimate of G for each group if all point patterns within a group are assumed to be realisations of point processes with the same intensity. Figure S34 shows the resulting curves, which do not show much difference between groups.

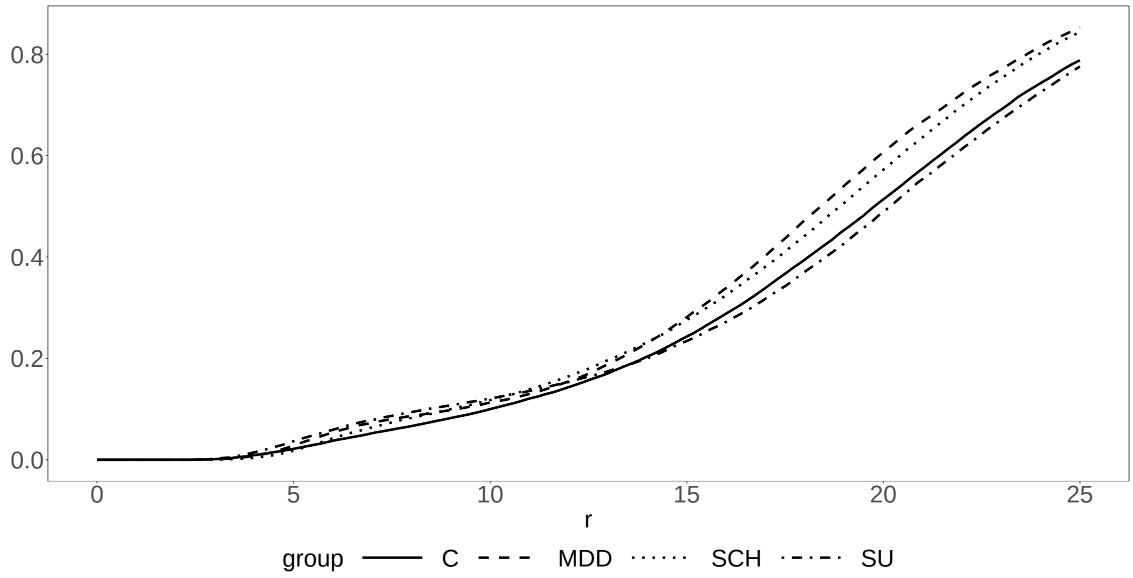

Figure S34: Weighted mean of the estimates of the G-function for each group as specified in the legend. Control (C), Major depressive disorder (MDD), Suicide (SU), and Schizophrenia (SCH).

To test whether there are significant differences in the G-function between the groups, we again used a 95% global envelope test in the same way as explained for the cylindrical K-function. The result can be seen in Figure S35, which shows no significant differences.

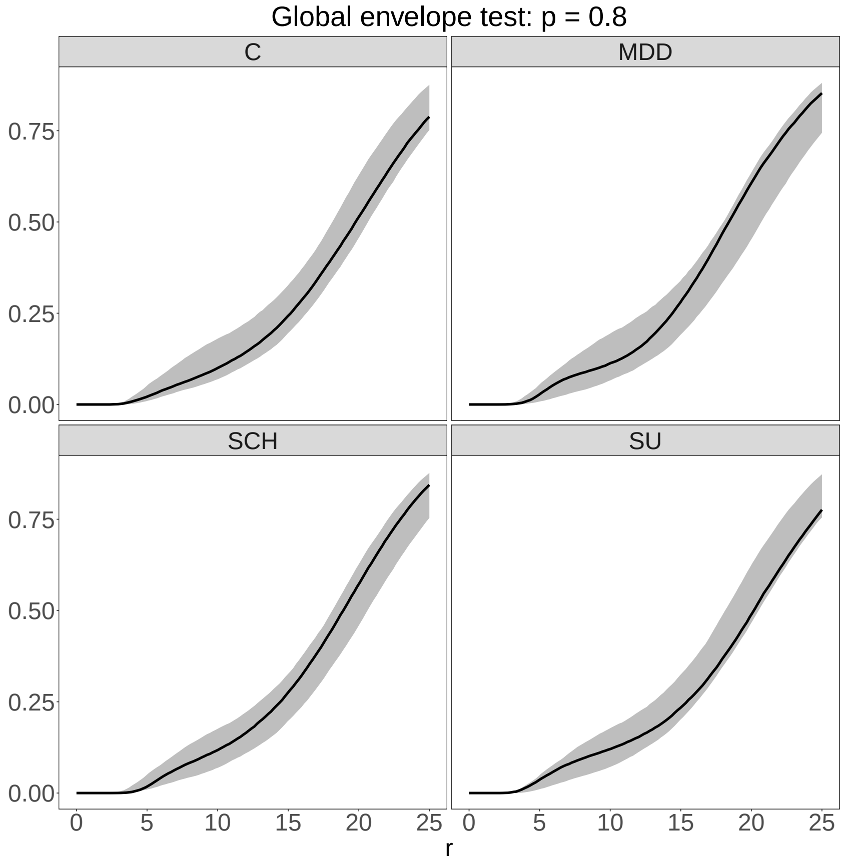

Figure S35: 95% global envelopes (gray area) based on 8000 random permutations for testing whether there are significant differences between the G-functions. The solid curves correspond to the data. The group is indicated at the top of each plot, and the p-value of the test is stated at the top of the figure. Control (C), Major depressive disorder (MDD), Suicide (SU), and Schizophrenia (SCH).

In spatial point pattern analyses, it is also very common to consider the functional summary statistic $J(r) = (1-G(r))/(1-F(r))$, which is constantly equal to 1 in the case of CSR. This very simple expression under CSR makes it very easy to make visual comparisons between an estimate of J and the theoretical value under CSR. However, we do not consider this summary statistic for the following reason. The estimation method used to estimate F for a 3D point pattern gives an approximation to $F(r)$ which should not be compared to its theoretical value under CSR [see 9]. Since the estimate of F does not behave as its theoretical value under CSR, neither will the estimate of J and we thus lose the easy visual comparison with CSR. Therefore, we do not consider J.
